# Patient and public involvement in Nordic healthcare research: a scoping review of contemporary practice

**DOI:** 10.1186/s40900-023-00490-x

**Published:** 2023-08-30

**Authors:** Kristine Elberg Dengsø, Sofie Tscherning Lindholm, Suzanne Forsyth Herling, Maja Pedersen, Kristina Holmegaard Nørskov, Marie Oxenbøll Collet, Iben Husted Nielsen, Mille Guldager Christiansen, Mette Schaufuss Engedal, Helga Wallin Moen, Karin Piil, Ingrid Egerod, Mogens Hørder, Mary Jarden

**Affiliations:** 1 Department of Surgical Gastroenterology, Copenhagen University Hospital, Rigshospitalet, Blegdamsvej 9, 2100 Copenhagen Ø, Denmark; 2 Department of Clinical Medicine, University of Copenhagen, Copenhagen Ø, Denmark; 3 Centre for Cancer and Organ Diseases, Copenhagen University Hospital, Rigshospitalet, Copenhagen Ø, Denmark; 4 Neuroscience Centre, Copenhagen University Hospital, Rigshospitalet, Copenhagen Ø, Denmark; 5 Department of Hematology, Copenhagen University Hospital, Rigshospitalet, Copenhagen Ø, Denmark; 6 Department of Intensive Care, Copenhagen University Hospital, Rigshospitalet, Copenhagen Ø, Denmark; 7 Department of Oncology, Copenhagen University Hospital, Rigshospitalet, Copenhagen Ø, Denmark; 8 Centre of Diaconia and Professional Practice, VID Specialized University, Oslo, Norway; 9 Department of Public Health, Aarhus University, Aarhus, Denmark; 10 Department of Public Health, University of Southern Denmark, Odense, Denmark; 11 Department of Hematology, Centre for Cancer and Organ Diseases, Copenhagen University Hospital, Rigshospitalet, Copenhagen Ø, Denmark

## Abstract

**Background:**

Over the past decades, there has been a growing international interest in user involvement in healthcare research. However, evidence on the management and impact of patient and public involvement in Nordic healthcare research remains limited.

**Objective:**

The aim was to explore and delineate the current state, practice, and impact of patient and public involvement in healthcare research across different areas of healthcare and patient populations in the Nordic countries.

**Methods:**

We conducted a scoping review using nine scientific databases and gray literature from 1992–2023. Sources were categorized as empirical or non-empirical. We used the Guidance for Reporting Involvement of Patients and the Public Short Form 2 checklist for reporting of patient and public involvement in healthcare research and the Preferred Reporting Items for Systematic reviews and Meta-Analyses extension for Scoping Reviews.

**Results:**

A total of 56 publications were included, consisting of 39 empirical and 17 non-empirical sources. Gray literature varied among countries and institutions encompassing different types of documents. We found an increase in the number of publications on patient and public involvement in Nordic healthcare research. This was evidenced by the growing number of references and institutional initiatives intended at involving the public, indicating the increasing emphasis on patient and public involvement in Nordic healthcare research. The terminology used to describe patient and public involvement varied over time. However, there has been a gradual narrowing down of terms as the concept of PPI has become more integrated into research practices, particularly with the involvement of funding agencies.

**Conclusion:**

The utilization of patient and public involvement in Nordic healthcare research has substantially increased, proliferated, and gained widespread acceptance across diverse healthcare domains. The variety of approaches challenged our scoping review in terms of systematic description and impact. Patient and public involvement was applied in one or more research stages using different methodologies and terms. International agreement on terms and definitions is needed for reliable interpretation of the use of patient and public involvement in Nordic healthcare research.

**Supplementary Information:**

The online version contains supplementary material available at 10.1186/s40900-023-00490-x.

## Abstract (plain language summary)

Over the past decades the importance of involving patients and the public as active partners in healthcare research has received growing acknowledgement internationally. Nonetheless, our knowledge regarding the degree of patient and public involvement (PPI) in the Nordic countries remains limited. This paper addresses this gap by investigating the status, management, and influence of PPI in healthcare research within the Nordic countries. The review of these aspects has given us a better understanding of PPI and its effects on healthcare research in the Nordic region. We looked at scientific databases and webpages including research papers, commentaries, and other materials from Denmark, Norway, Sweden, Finland, and Iceland. Our goal was to gather information and provide a thorough overview of PPI practices. Our findings showed that PPI is growing with gained acceptance across different areas of health research. PPI was used at different stages of the research process, but there wasn’t a common agreement on its importance and the additional value it brings to the quality of research. The study was challenged by the many different terms and definitions, which affected the clarity of our study’s purpose (or goals).However, we made efforts to address this by carefully reviewing the different terms and definitions used in the literature, striving to capture the essence of PPI in our analysis. By acknowledging this variation, we aimed to provide a wide-ranging overview while identifying the complexities and nuances related with PPI in the Nordic healthcare research. We assume that achieving international agreement on terms and definitions of PPI would certainly improve the trustworthiness in future reviews.

**Supplementary Information:**

The online version contains supplementary material available at 10.1186/s40900-023-00490-x.

## Background

Patient and public involvement (PPI) has become increasingly integrated in research internationally, especially in Europe and North America [1, 2]. In Europe, The United Kingdom (UK) has been on the forefront of this movement, followed by the Netherlands and Scandinavian countries (Biddle et al. 2021). People involved, other than the research team, in the research process is gaining momentum. In this context, the people involved refer to patients and public contributors who are invited to engage in healthcare research. Of importance is the inclusion of patients and their families as partners in the research process [1]. As such, patients and families have evolved from being solely the subjects of research to actively participating as partners throughout the research process [3].

PPI is increasingly being recognized by funding agencies and patient organizations as an integral part of healthcare research [2, 4, 5]. Prominent UK models have taken leading roles in the field of PPI, including the James Lind Alliance (JLA) and Priority Setting Partnerships (PSPs) where collaboration among patients, families, and clinicians is essential in order to identify research priorities [3]. Moreover, the government funded National Institute for Health and Care Research (NIHR) has played a pivotal role in advancing PPI [6–9]. Established in 2006, NIHR categorize the research process into seven stages which include identifying and prioritizing, commissioning, designing and managing, undertaking, disseminating, implementing and evaluating impact [7]. Further, NIHR uses terms such as consultation, collaboration, co-production, and user control to define varying degrees of involvement. NIHR has made substantial global impact on research and healthcare, significantly contributing to the improvement of treatment and care [10]. These initiatives closely align with the progressive and contemporary healthcare systems present in the Nordic Countries.

Earlier, PPI has been criticized for exclusivity and tokenism reducing patient stakeholders to a perfunctory role in healthcare involvement [1, 8, 9].Currently, people involved are increasingly recognized and valued as integral members of the research team [11, 12].

According to Engelstad et al., the Nordic societies represent a common neo-corporatist model characterized by a strong and active state, a high degree of labor market coordination and a comprehensive welfare state [13]. PPI is suggested to increase the cost effectiveness of research by ensuring that research outputs align with the patient groups’ needs [14].Evaluating the impact of PPI remains limited to involved patients and researchers, often neglecting to specify how PPI influenced study outcomes [15]. There is a gap in understanding how PPI is integrated into the research process, including the utilization of PPI terms, methodologies as sampling strategy and theoretical frameworks and its effects on study outcome in the Nordic countries of Denmark, Norway, Sweden, Finland, and Iceland. These countries share cultural values and healthcare systems, facilitating research collaboration. The aim of this scoping review is to explore and delineate the current state, practice, and impact of PPI in healthcare research across different specialties and patient populations within the Nordic countries.

## Methods

The scoping review and search strategy was based on Preferred Reporting Items for Systematic Reviews and Meta-Analyses (PRISMA) with the extension for scoping reviews [16, 17] and registered at the Research Registry (Identifier: research registry 7157) on September 16, 2021 [18]). We followed the five steps for conducting scoping reviews outlined by Arksey and O'Malley [19].

### Step 1 identification of research questions

The research team devised the following research questions for references reporting PPI in healthcare research:

1. How extensively is PPI reported in healthcare research across the five Nordic countries? This includes its origin, document type, population and the represented perspectives.
2. What theoretical frameworks are utilized to characterize PPI?
3. During which stages of the research process and by means of which methods is PPI incorporated?
4. What are the positive and negative impacts of PPI reported in the literature?

### Step 2 identification of relevant references

A systematic search was conducted across nine scientific databases to identify papers published between January 1992 and April 2023. We included empirical studies comprising quantitative, qualitative, multiple method studies or mixed methods research. The non-empirical papers included were protocols, discussion, description, and perspective papers. We searched the following bibliographic online databases: Cochrane Library (Cochrane Central Register of Controlled Trials (CENTRAL), Excerpta Medica Database (EMBASE), Cumulative Index to Nursing and Allied Health Literature (CINAHL), Medical Literature Analysis and Retrieval System Online (MEDLINE), PsycINFO, Allied and Complimentary Medicine Database (AMED), SCOPUS, Sociological abstract and Web of Science. Our search matrix included a modified PICO (patient, intervention, comparison, outcome) framework to structure clinical research questions that are important for the patient or population [20]. Further, PICO was also used to formulate the search strategy by identifying key concepts. This framework defined the population, intervention, and context within our primary focus areas: 1) Patient and public involvement, 2) Health research, and 3) Nordic countries. A combination of MeSH-terms, Thesaurus, indexed terms, and free text were used. An example of a search strategy from Medline is provided in Additional file 1. The following MeSH search terms were used within each of the three focus areas: (1) PPI: Patient partner, participant, consumer, user, public, patient participation, community participation, panel, advisory group, engagement, participation, collaboration. (2) Health research: Healthcare research in all care sectors, clinical research. (3) Nordic countries: Denmark, Norway, Sweden, Finland, and Iceland. Language limitations: English, Danish, Norwegian, and Swedish. Language exclusion: Finnish and Icelandic.

To identify gray literature, we conducted a systematic search of webpages for policy documents, reports, academic papers and documents, course materials, events, recommendations etc. from ministries, universities, patient organizations, funding bodies, and healthcare research platforms. Additionally, we contacted researchers from each Nordic country to help identify additional gray literature sources. Supplementary searches were carried out using manual and snowball search methods.

### Step 3 selection

To comprehensively address the concept of PPI, we included references if they utilized, clarified or emphasized PPI within healthcare research across any setting in one or more Nordic countries. We included all papers irrespective of the PPI terminology used to refer to ‘people involved’ participants. However, we excluded papers that used PPI within a treatment context rather than within research. Three investigators, KD, SFH and STL individually screened the identified references and determined inclusion. KD, SFH and STL individually excluded non-relevant references by reviewing titles and abstracts. References identified as relevant by either reviewer underwent full text reading by KD and STL. The references were categorized as “included”, “unclear” (for discussion) or “excluded”. In case of disagreement a fourth senior researcher (MJ) provided consultation. Covidence software program (2023) was used for screening process [21].

### Step 4 chart and extract the data

Papers from the systematic search in Covidence were extracted by using the NIHR framework (identifying and prioritizing research, commissioning research, designing and managing research, undertaking the research, disseminating research, implementing research, and evaluating impact) was additionally used in the extraction [6] and the Guidance for Reporting Involvement of Patients and the Public Short Form (GRIPP2-SF) [22]. We used the short version of the guidance (GRIPP2-SF), which can be used when reporting public involvement in any study, rather than  the long version (GRIPP2-LF), which is used when the study is mainly about public involvement in research [23]. We therefore modified to include additional categories: type of document, study design, study population, PPI term, participants, theoretical underpinning. Table 1 provides an overview of the modified GRIPP2-SF. To refine the modified GRIPP2-SF three papers were pilot tested prior to final inclusion. The content of the included references was assessed according to the modified GRIPP2-SF by KD and STL individually before consensus decisions were made. In case of disagreement discussions were held with MJ to reach resolution.

**Table 1:** Adapted guidance for reporting patient and public involvement, modified guidance for reporting involvement of patients and the public short form

Section and topic	Item
Author/year of publication	
Aim	Report the aim of PPI
Type of document^a*^	
Study design^a^	Qualitative or quantitative design
Study population^a^	Which patient or public population took part in PPI
PPI term¶	Term used to describe PPI
People involved	People involved in PPI
Theoretical underpinning^a°^	The theoretical rationale and any theoretical influences relating to PPI
Identifying and Prioritizing^a^	NIHR
Commissioning^a^	
Designing and managing^a^	
Undertaking^a^	
Disseminating^a^	
Implementing^a^	
Evaluating Impact^a^	
Method used	
Results	Outcomes: Report the results of PPI in the document, including both positive and negative outcomes
Methods to evaluate impact	
Findings	The impact of PPI on researchers, patients and public involved in the research process
Discussion and Conclusions	Outcomes: Comment on the extent to which PPI influenced the study overall. Describe positive and negative effects
Reflections	Comment critically on the study, reflecting on the things that went well and those that did not, so others can learn from this experience

^a^Adjusted sections from GRIPP2-SF

*Visualized in Fig. 2

°Visualized in Table 4

^¶^Described in text

### Step 5 collate, summarize, and report the results

We present the results in a narrative format, supplemented with tables and figures, and organized to address the five research questions. In addition, the findings are categorized according to reference type, including research studies, non-empirical papers, and gray literature sources. If terms of PPI participants being used appear similar, the research group discussed how to summarize the terms to create a more simplistic overview. We defined public involvement as anyone else being involved than patients, e.g. families, cares, healthcare researchers and other stakeholders.

## Results

**Fig. 1:**
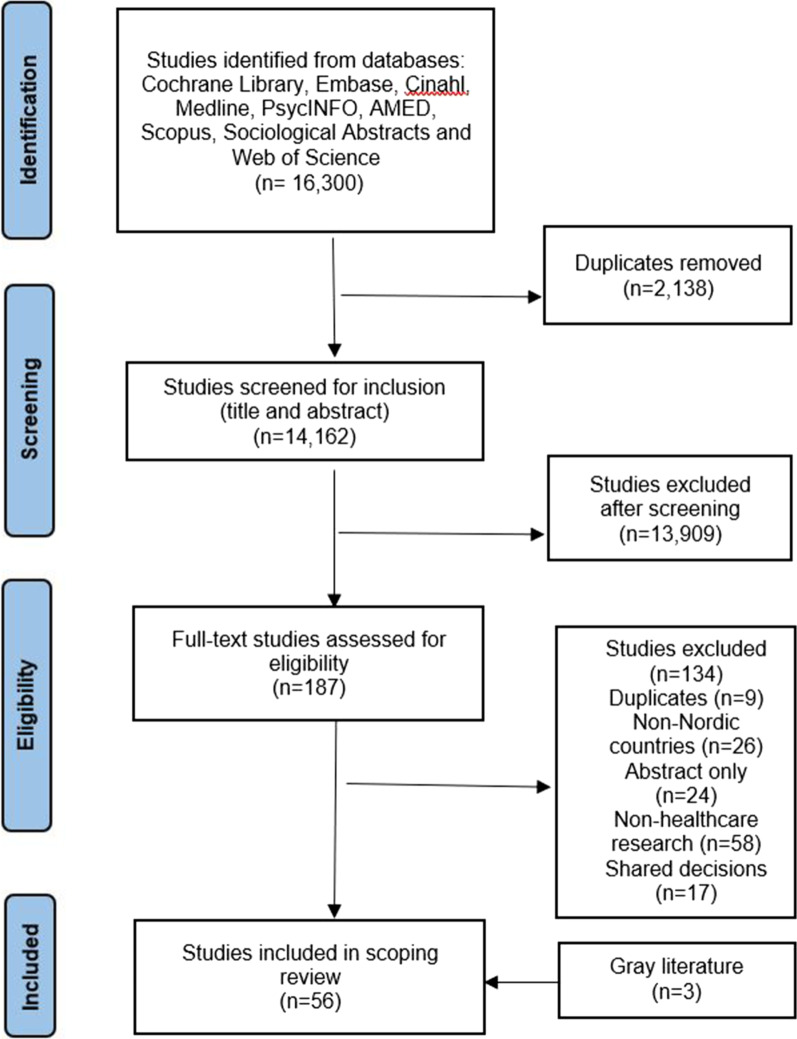
PRISMA flowchart visualizing the overview of the inclusion and exclusion of papers in the systematic screening process

We included 53 papers from the scientific databases and three papers from gray literature, incorporating a total of 56 papers in this review as illustrated in Fig. 1. All 56 papers are presented in Table 2. Further, Table 3 displays policy documents and other non- scientific sources that were identified through gray literature searches. In the following section, we provide an overview of the types of evidence, documents, and other materials available regarding PPI in healthcare research in the Nordic countries.

**Table 2:** Characteristics of the included papers (n = 56) from the systematic literature search extracted with the use of a modified GRIPP2-SF

Author/year	Aim	Type of document	Sampling strategy	Study population	People involved	Identifying and Prioritising	Commissioning	Designing and Managing	Undertaking
*Denmark*									
Timm et al. (2022)		Empirical	NA	Gestationel diabetes mellitus	Women with prior gestationel diabetes mellitus and their families	NA	NA	Involvement practices were adapted on an ongoing basis to secure meaningful engagement	NA
Christiansen et al. (2022)	To develop a model for systematic nurse-led consultations based on ePRO	Empirical	NA	Ovarian or endometrial cancer	Women with history of ovarian or endometrial cancer	NA	NA	Feedback on different materials	NA
Bundgaard et al. (2022)	To initiate the debate about PPI	Non-empirical	NA	NA	NA	NA	NA	NA	NA
Thomsen et al. (2022)	Describe the development through participatory design of a comprehensive transfer program targeted to parents of adolecents with chronic illness	Empirical	NA	Young people with chronic illness	Parents and young people with affiliation to outpatient clinics	NA	NA	Gave input to the intervention	NA
Kjær et al. (2021)	To delvelop a core outcome set for intensive care unit patients	Empirical	NA	Intensive Care Units	Former intensive care patients and relatives	NA	NA	NA	NA
Missel et al. (2021)	To explore patients' and spouses' perception and attitudes towards participating in a patient and family advisory council	Empirical	Convenience sampling	Relation to Dept. of Cardiothoracic and Vascular Surgery	Former patients and/or spouses	NA	NA	NA	NA
Berring et al. (2021)	To learn about dynamics in a small group collaborative process influenced the the establishment of a research partnership	Empirical	NA	Persons who has attempted or who has died by suicide	Members of the cooperative inquiry group with experience of elder person who have died by suicide	Idea generation	NA	Formulated interview questions	NA
Barot et al. (2021)	To investigate the inclusion of PPI in contemporary, large RCTs conducted in the ICU setting	Non-empirical	NA	Intensive Care Units	NA	NA	NA	NA	NA
Hansen et al. (2021)	To investigate the extent to which patients and relatives were willing and able to be involved as fellow transitional care researchers while seeking relevant transitional care outcome measures and investigating patients’ views on care transitions	Empirical	Convenience sampling	Frail older patients	Frail older patients, relatives and HCPs	Identifying outcome measures for future research	NA	NA	NA
Finderup et al. (2021)	To describe how patients were involved in a research project; To explain what occurred when patients were involved; To identify facilitators of and barriers to patient involvement in research and transform these into practical recommendations	Empirical	NA	Chronic kidney disease	Patients who participated in the final two years of the research case	Identifying and prioritizing research questions	Recrutiment through the Danish Kidney Association	Support planning of the project	Interpretation of findings
Kirk et al. (2021)	To explore and discuss the key challenges associated with having stakeholders take part in the design of a health care intervention	Empirical	Maximum variation	Older patients	Health professionals, patients and relatives	NA	NA	Design the study process	NA
Skovlund et al. (2020)	To explore ways to embrace the perspectives of patients in a research process, and the impact and challenges of collaboration on patients, researchers, and the research outcomes	Empirical	NA	Metastatic melanoma	Patient representatives	Prioritizing research questions	Choosing projects to fund	Choose PRO-measures for the dialogue tool and the research project, to compose patient information sheet, and to decide on a relevant design	Comprised a joint training day for researchers and PRPs, two consensus meetings, at which the codes were discussed, based on individual code-work done as homework, and an evaluation workshop
Høeg et al. (2019)	To examine how involving patients with lower levels of education affected PPI in the development of a clinical trial from the perspectives of the patients, recruiting nurses and researchers involved	Empirical	NA	Breast cancer	Patients who had completed breast cancer treatment	NA	NA	NA	NA
Beedholm et al. (2019)	To contribute to the approach dealing with contextual and structural factors of significance for patient involvement by demonstrating how inspiration from institutional theory widens our insights into the challenges of changing a hospital into a “patient involving hospital.”	Non-empirical	NA	NA	NA	NA	NA	NA	NA
Piil et al. (2019)	To identify future research agendas that reflect the concerns and unexplored areas of interest for patients with life‐threatening cancer, their relatives and the clinical specialists during the cancer trajectory	Empirical	NA	Life‐threatening cancer	Patients, relatives and clinical specialists	Identifying future research agendas	Danish Brain Tumour Organisation and patient support organisation for lymphoma and leukaemia	NA	NA
Nissen et al. (2018)	To present researchers experiences from a shared working group with patient representatives and researchers	empirical	NA	Breast or prostate cancer	Two patient representaives and researchers	NA	NA	Evaluation of interviews and discussion of program material	NA
Jørgensen et al. (2018)	To investigate the impact of involving patient representatives as peer interviewers in a research project on patient empowerment	Empirical	NA	Cancer follow-up care	Patients and patient representatives as peer interviewers	Workshop to discuss the proposal	NA	To form the development of th ePROM questionnaire	Interviewed peers
Handberg et al. (2017)	To perform a secondary analysis discussing clinical and methodological aspects of patient involvement in clinical research and practice by investigating perspectives of patients and healthcare professionals on fertility treatment	Empirical	NA	Fertility treatment	Women undergoing fertility treatment	Prioritising research questions	NA	NA	NA
Piil et al. (2016)	To identify, discuss and prioritise future research issues within supportive care and rehabilitation in patients with primary malignant brain tumours and acute leukaemia during the cancer trajectory	Non-empirical	NA	Primary malignant brain tumours and acute leukaemia	Patients, relatives and specialists	Prioritising research questions	Representatives from frelevant patient organizations	NA	NA
Madsen et al. (2015)	To develop an understanding of how men experience ankylosing spondylitis	Empirical	Purposive sampling	Ankylosing spondylitis	One male patient diagnosed with rheumatic disease	Generation of ideas	NA	Development of interview guide	Feedback on the written patient informaiton sheet
Norway									
Solbakken et al. (2022)	To identify a pragmatic priority setting process to identify a prioritized top 10 list of research needs	Empirical	Convinience/snowball sampling	Stroke	Patients with stroke	NA	NA	NA	NA
Gilhus et al. (2022)	The aim for patient involvement in Myasthenia Gravis research is to improve quality, increase research output and relevance, dissemination of results and secure implementation	Non—empirical	NA	Myasthenia Gravis	NA	NA	NA	NA	NA
Jokstad et al. 2022	To describe and reflect on the process and outcomes associated with advisory group-researcher collaboration from a person-centered approach	Empirical	NA	Older adults with health-related conditions	Older adults	Prioritising research questions	Older adults with health related conditions	The participants were asked to evaluare the proposed design of the study and were asked about their views of the guestion guide	NA
Guise et al. (2021)	To investigate how involvement of patients and stakeholders in recilience in health care is described and improves	Non-empirical	NA	NA	Patients and other stakeholders	NA	Different patients groups	NA	NA
Stuhlfauth et al. (2021)	To explore how guidelines construct the perception of users and researchers and thus the process of involvement	Empirical	NA	NA	NA	NA	NA	NA	NA
Koren Solvang et al. (2021)	To determine what knowledge types and competences users apply when involved in the research process through user panel meeting	Empirical	NA	Rehabilitation service users	Rehabilitation service users and researchers	NA	Two disability nongovernmental organizations were contacted and asked to recruit panel members	Discussion of interview guide	Selected interview transcrips were discussed
Slåtsveen et al. (2021)	To describe a way of applying NLR in a small-scale study and to address strengths and limitations of this way of employing user involvement	Empirical	NA	NA	Clinicians, organisations for service users and next of kin, members of senior citizens’ councils and the Patient Ombudsman	User involvement to devise research relevant questions for clinicians, service users and next of kin	Organisations for service users and next of kin, members of senior citizens’ councils and the Patient Ombudsman were invited to a workshop	NA	Themes were discussen and reorganized
Sand et al. (2020)	NA	Non-empirical	NA	NA	NA	NA	NA	NA	NA
Staats et al. (2020)	To formulate a framework for patient and informal caregiver participation in research— replacing the old focus of “them” as research objects, with focus on “us” as fellow researchers	Non-empirical	NA	Vulnerable people experiencing incurable life-threathening illness	Patients and informal caregivers	NA	NA	NA	NA
Stuhlfauth et al. (2020)	To explore and describe how equity is constructed through the emerging storylines that users and researchers draw upon	Empirical	NA	NA	Users and researchers experienced in user involvement in research	NA	NA	NA	NA
Stuhlfauth et al. (2019)	To investigate experiences and collaboration between patient representatives and researchers in user involvement in health research	Empirical	Snowball sampling	Disabled people	Researchers and patient representatives	Identifying reseach questions To lay the grounds for participation in the development project to discuss previous experiences from involvement in research through focus groups	NA	NA	NA
Mjøsund et al. (2018)	To explore the process of involving mental healthcare research advisors in a mental health promotion project and to articulate features of the collaboration that encouraged and empowered the research advisors to make significant contributions to the research process and outcome	Empirical	Purposive sampling	Severe mental illness	Researchers and the research advisors	NA	Application for funding	Design of methodology	Analysis of data
Mjøsund et al. (2017)	To examine how service user involvement may contribute to the development of IPA (Interpretative Phenomenological Analysis) methodology and in turn enhance the research quality	Empirical	NA	Severe mental illness	Five research advisors either with a diagnosis or related to a person with severe mental illness constituted the team	NA	NA	Advisory board of patients and relatives	NA
Natland et al. (2017)	NA	Non-empirical	NA	NA	NA	NA	NA	NA	NA
Tangvald-Pedersen et al. (2017)	To present an overview of three methodological standards and their respective dependency on three different ideologies or voices that advocate for user involvement and participant research; and to share experiences of designing a solution given these three voices	Non-empirical	NA	NA	NA	NA	NA	NA	NA
Moltu et al. (2013)	To explore how service users experience their participation as coresearchers in ongoing mental health research projects and how their attendance at a training program in research methodology is experienced to influence this collaboration	Empirical	Purposeful sampling	Mental health service users	Coresearchers with service user background who had an interest in contributing to research and who had experienced mood disorders and recovery	NA	Patients with interest in participating in research	NA	The preliminary analyses back to the participants for discussion, auditing, and reanalysis
Moltu et al. (2012)	To examine coresearchers lived experiences of what it is like approaching the academic world from a service user perspective	Empirical	Purposeful sampling	Mental health service users	Mental health service user who had an interest in contributing to research and who had lived experiences of mood disorders as well as recovery processes	NA	NA	NA	Discussion, auditing, and reanalysis. Themes were reorganized
Kjeken et al. (2010)	Describe the research priorities of people in Scandinavia, their experiences and attitudes concerning participation in research projects, and which format for research information they prefer	Non-empirical	NA	Rheumatic diseases	Patients	NA	NA	NA	NA
*Sweden*									
Frögren et al. (2022)	To investigate the awareness of and attitudes towards public involvement in research on older people	Empirical	NA	Older people	Elderly people from a previos study	NA	NA	NA	NA
Siira et al. (2022)	Description of online citizen panels	Non-empirical	NA	Cardiovascular diseases	NA	NA	NA	NA	NA
Nyman et al. (2022)	To describe the process of using participatory action research	Empirical	NA	Rehabilitation	Patients undergoing home based rehabilitation	Deliniate the potential problems for research	NA	Discussing different solution for the intervention	NA
Schandl et al. (2022)	To describe and evaluate the development of PPI in oesophageal cancer survivorship	Empirical	NA	Oesophageal cancer survivors	Mostly elderly men with cancer	Identifying purpose and setting	Gave perspectives on grant applications	Gave perspectives on study design	Interpretation of results
Rudberg et al. (2021)	To determine which areas of research related to life after stroke that Swedish stroke patients and their informal carers consider to be relevant and valuable	Empirical	NA	Stroke	Patients and relatives	Prioritising research questions	NA	NA	NA
Berge et al. (2020)	Explore frail old peoples experiences of involvement in research	Empirical	NA	Physically frail older people	Older people with experience of participating in randomized controlled studies	NA	NA	NA	NA
Kylén et al. (2020)	To enhance the execution of high-quality research and to increase the knowledge about the added value stemming from user involvement in the research process	Non-empirical	Convenience sampling	Older people	People aged 60 years and older ; Informal caregivers ; Professionals within health care and architecture Researchers in aging and health	NA	Carer recruited from non gowermental organizations	NA	NA
Warner et al. (2019)	To describe the group dynamic characteristics and impact of PPI from the user perspective in a case study of refugee involvement in health research	Empirical	NA	Mental health	Refugees with post traumatic stress	The refugee advisors discussed ideas of research with the researchers	NA	Gave impact to the designings and managing process by diskussing differrent alternatoves to the proposed design etc	NA
Iwarsson et al. (2019)	NA	Non-empirical	Stratified sampling	Ageing people	Ageing people	NA	NA	NA	NA
Acosta et al. (2019)	To establish the top 10 research uncertainties in AD using the JLA concept	Empirical	NA	Older people with disabilities	Patients and HCPs	Prioritising research questions	ADAS members and caregivers	NA	NA
Kylén et al. (2022)	To examine researchers experiences and perspectives of user involvement in research on aging paople	Empirical	NA	Older people	Older people from the personal address register	NA	NA	NA	NA
Kumlien et al. (2022)	To establish a priority setting partnership between participants and clinicians to identify the top 10 research priorities for preventing and treating diabetic foot ulcers	Empirical	NA	Diabetes mellitus	Participants living with diabetes mellitus and clinicians	Finding and prioritising research priorities	NA	NA	NA
Malm et al. (2022)	To explore researchers' views of involving informal carers in health and social research	Empirical	NA	Disabled people	Researchers	NA	NA	NA	NA
Malm et al. (2019)	To explore how carers perceived and reflected on carer involvement in Research and Development work, with specific reference to their personal experiences of being involved in the development of a Swedish carer strategy	Empirical	NA	Older people with disabilities	Informal carers	NA	Representatives from carer and patient organizations	NA	NA
Bergsten et al. (2014)	To follow the working process of involving patients in a project group and to describe the research issues that were important from the patient’s point of view	Non-empirical	NA	Rheumatic diseases	Patients	Identification of research ideas	National patient organizations and research and development center in joint project	NA	NA
Carlsson et al. (2006)	To explore PACP members’ and health care professionals’ experiences of collaboration	Empirical	NA	Cancer	Members of patient cancer associations and health care professionals	NA	NA	NA	NA
*Finland*									
Jones et al. (2020)	To explore how people become involved and how they construct the accounts of their lived experiences	Empirical	N/A	Mental illness	Patients living with mental illness	NA	NA	NA	NA
Jones et al. (2017)	To describe and analyse the development of patient and public involvement from a policy perspective	Empirical	NA	NA	NA	NA	NA	NA	NA

Author/year	Dissiminating	Implementing	Evaluating Impact	Methods used	Results	Methods to evaluate impact	Findings	Discussion and Conclusions	Reflections
*Denmark*									
Timm et al. (2022)	NA	NA	NA	Interviews	Involvement was sometimes percieved too timeconsuming and burdensome especially in thoses cases where the stakeholders suggestions were not adpoted	NA	NA	There is a need to further document the developmental work of intervention research	NA
Christiansen et al. (2022)	NA	NA	NA	Advisory board	NA	NA	NA	NA	NA
Bundgaard et al. (2022)	NA	NA	NA	NA	NA	NA	NA	NA	NA
Thomsen et al. (2022)	NA	NA	NA	Interviews and workshops	NA	NA	NA	By incorporating the principles of particapatory design in the development phase the authors ensured that both parents and adolecents needs were represented and met in the program	
Kjær et al. (2021)	NA	NA	NA	NA	NA	NA	NA	NA	NA
Missel et al. (2021)	NA	NA	NA	Focus group interviews	Patients participatied in advisory boards because of Payback, personal invitation, safe and equal atmosphere and Sharing, caring, and healing	Focus groups	Participants described the inclusion in the council as an opportunity for them to thank and payback to the health-care professionals and the system for treating them when they were most in need	After undergoing surgery, patients and spouses express a need for “paying it back” to the health-care system by participating in an advisory council. When being part of this, the participants expressed feeling a genuine engagement and interest from the health-care professionals. They expressed how this leads to a feeling of being equal and taken seriously, though it cannot be ruled out that power differentials in the advisory council affected what the participants were willing to say	NA
Berring et al. (2021)	NA	NA	NA	Interviews	NA	NA	The results highlight that a genuine partnership can grow out of a cooperative inquiryif all members of the group contribute equally to the research	The study shows how human flourishing grew out of the process of co-creating the interview guide and user-researchers became empowered change agents	NA
Barot et al. (2021)	NA	NA	NA	NA	NA	NA	NA	NA	NA
Hansen et al. (2021)	NA	NA	NA	Individual interviews Panel-based discussions	NA	HCPIC Health Canada Public Involvement Continuum	Patients ere involved in discussing care transitions (HCPIC level 3), while some relatives were engaged (HCPIC level 4) in forming PROMs. The partnership level of involvement (HCPIC level 5) was not reached	When applying a pragmatic involvement approach, frail older patients can be successfully involved in identifying relevant transitional care outcome measures; however, involving patients as fellow researchers seems infeasible. To maintain involvement, supportive relatives are essential	Involvement of patients, relatives and other stakeholders holds the potential to become an inherent and valuable part in geriatric, frailty and transitional care research studies
Finderup et al. (2021)	NA	Ensuring the results were applicable to clinical practice	NA	Semistructured individual interviews	Eight facilitators and barriers were identified	Individual interviews	Patients experienced a sense of meaningful contribution to the research project The patient is not only a giver but also a receiver Both qualitative and quantitative studies as well as the SDM‐DC intervention benefitted from patient involvement. The patients themselves also benefitted from their involvement in the research. Important facilitators of involvement of patients with CKD in research include working as a team, being a part of a process, and being prepared for the work	Patients perceived themselves to be both givers and receivers who contributed to the research project but also gained something from the project. Patients experienced a sense of equal teamwork with the clinicians and researchers	Some phases of the research project with possibilities for more involvement. Neither of the patients thought that they could be a more active part of recruitment of participants and data collection than they already were. Important barriers to patient involvement include patient vulnerability
Kirk et al. (2021)	NA	NA	NA	Workshops	Two themes emerged: A; Engagement refers to different challenges in recruiting stakeholders in the co-design process. B; Facilitation refers to different challenges for the research team with regard to changes in roles and activities. The theme engagement consists of two sub-themes: recruiting patients and involving physicians. Facilitation consists of three sub-themes: adjusting to a new researcher role; utilizing contextual knowledge and handling ethical dilemmas	NA	NA	Two key challenges associated with having health professionals, patients and relatives co-designing an intervention to increase mobility in older medical patients admitted to a hospital in Denmark were idenyified. The challenges were related to engagement and facilitation	It is not only patients and relatives who need to be prepared to be part of stakeholder engagement and design processes. Researchers who want to use co-design must be prepared for the extra time required and the need for “engagement literacy”, that is, skills concerning communication, facilitation, negotiating and resolving conflict
Skovlund et al. (2020)	Meetings were held between PPI and one patient representative. This patient had been awarded a PRP scholarship to attend a conference together with the PI to present the work and thoughts on PPI. This PRP also engaged in the writing of the present article	Feed back from clinicians and discussions about implementing plan	Participate in the evaluation of clinical impact	Multiple sources of data: email correspondences, sticky notes, coding schemes, records of the proceedings, discussion-notes, and audio recordings from the evaluation workshop	The patients contributed with a new vocabulary and perspective on the dialogue, and they validated the results PPI caused considerations related to emotional (sadness/sorrow and existential thoughts), administrative (e.g. arranging meetings, balancing work and small talk) and intellectual (e.g. avoiding information harm, continuing activities despite the death of patients) investments	NA	The impact of PPI on PRPs, researchers and research outcome was qualitatively explored through all the above-mentioned sources of data, particularly from consensus on records of the proceedings and the workshop A limitation of the study was the lack of use of a solid evaluation tool to determine the impact of PPI	NA	NA
Høeg et al. (2019)	NA	NA	NA	Focus group interviews Individual interviews	Patient feedback led to changes and improvements in recruitment strategy, brochures and educational material, the electronic platform created to collect questionnaire data, as well as helping researchers ensure that questionnaire items were generally understandable and not offensive. However, changes were not made to item wordings or answer categories in order to uphold the validation of the scales. In this aspect, the interests of research were privileged above those of the patients and we return to this in the discussion	The cube model	Our results highlight the complexities involved in integrating the patient perspective in the research process Successful patient involvement involves the dynamic interactionof patient and researcher knowledge, but this gives rise to many dilemmas	More specific guidance needs to be developed in collaboration with funders, researchers and patients, which includes how to manage the tensions between patient and expert priorities in specific research settings	This study has several limitations. The involvement of patients relatively late in the development of the trial and the use of semi-structured interview guides with predefined areas may have limited how researchers could use the patient input
Beedholm et al. (2019)	NA	NA	NA	NA	NA	NA	NA	The theoretical framework on institutional logics provided concepts that extended our understanding of the challenges related to the implementation of patient involvement methods The examples indicate that if patient involvement—in terms of the efforts to consider the patient's perspectives, wishes and needs to the same extent as other parameters in the healthcare system—becomes a reality, it requires an independent analytical concept, such as a “patient logic”	An appropriate model for the development of the healthcare system of the future should be expanded with a fifth logic, a “patient logic.” Future research should contribute with a differentiated description of the characteristics of such a logic, and how it is incorporated in the balance of power between the existing institutional logics
Piil et al. (2019)	NA	NA	NA	NA	New research agendas related to high‐grade glioma brain tumour and acute leukaemia with corresponding research questions were formulated within the topicsof supportive care/palliation, education/information, rehabilitation, complementary and alternative therapy and organization of health care	Likert scale evaluation	All participants fully agreed that they had shared the most important issues from their perspective, some elaborated on this and added that focus on caregivers and communication with the healthcare professionals is important aspects	User involvement within a qualitative approach can be a valuable method applied alone or together with Delphi studies and surveys in identifying research agenda User involvement in identifying research agendas has the potential to improve quality of care for patients and caregivers across the cancer trajectory, while minimizing the gap in research between the healthcare user and healthcare provider	A limitation was the small sample sizes
Nissen et al. (2018)	NA	NA	NA	NA	To present evaluation data material was collected from meeting documents, interviews and fieldnotes	Qualitative	The changes in the program and the research project were related to user firendly wording of text and procedures adjusted for the specific cancer population		Describe the importance of researchers being clear of the purpose of involving patients
Jørgensen et al. (2018)	NA	NA	No firm conclusions could be made about impact on outcomes	Interviews	Interviewees were generally content with the process of having a peer interviewer present, and some had felt that being interviewed by someone with similar experiences had been very useful	Individual interviews		There are good arguments for using peer interviewers in qualitative health research	It is important to consider potential benefits alongside relevant ethical considerations, available resources for support of both peer interviewers and interviewees, and the need for training, not only in interview techniques, but also in reflexivity and professional/personal boundary work
Handberg et al. (2017)	NA	NA	As a result of PPI a RCT was cancelled	Focus group interviews	it became apparent that the women exercised and maintained a clear perspective on their hope for a child, Project Child, while the interviewer pursued a treatment perspective, Project Treatment. Despite different perspectives, the conversation during the interviews seemed effortless, and it became apparent how the interviewer and the participants were actually focusing partly on the same, but primarily on different issues but without addressing or acknowledging this. Knowledge and awareness of the difference in perspectives is important when healthcare professionals seek to involve patients both in clinical practice and in research	Focus groups	The planned RCT study was canceled as a result of the focus group interviews and as such was a successful example that patient involvement can impact research designs	The study shed additional light on clinical and methodological aspects of patient involvement in clinical practice and research. Due to the logics and organization of healthcare, implementing patient involvement has been shown to be challenging. The kind of information and type of insight that can actually be obtained by asking patients about their perspectives, and how this information or insight may or may not strengthen the quality of research, treatment and care have not received extensive consideration	When involving patients, it is mandatory to take as the starting point the patients’ perspective and life world. Patient involvement is not achieved simply by inviting patients to participate in their treatment and care, and the research around it. It requires not only that healthcare professionals involve themselves in their patients’ everyday lives, but also that pathways are organized and decisions shared in a manner that promotes patient involvement in daily clinical practice A limitation that the data were not collected to address the particular research question of this article and that not all of the authors were involved in the initial data collection. This might have caused unawareness of study-specific nuances or glitches in the interviews that may be important to the overall interpretation. On the other hand, a new approach by new researchers may also ensure consistency and repeated discussion and validation of the findings, in line with the applied analytic methodology
Piil et al. (2016)	NA	NA	NA	Focus group interviews	NA	NA	NA	NA	NA
Madsen et al. (2015)	Dissimination of findings and commented the manus	NA	Enhanced and validated the study design from a patient perspective	NA	NA	NA	NA	NA	NA
Norway									
Solbakken et al. (2022)	NA	NA	NA	NA	NA	NA	NA	NA	The study highlights a prioritized top 10 list of research aims
Gilhus et al. (2022)	NA	NA	NA	NA	NA	NA	NA	NA	NA
Jokstad et al. 2022	NA	NA	NA	Focus groups	NA	NA	NA	NA	Valuable ideas and knowledge would have been lost if the researchers had not embraced the idea of user involvement in the research
Guise et al. (2021)	NA	NA	NA	NA	NA	NA	NA	NA	This is a protocol who have had PPI integrated in the funding process and have planned to include PPI throughout the whole research process
Stuhlfauth et al. (2021)	NA	NA	NA	Critical analysis of documents as guidelines	The analysis indicates that users and researchers are constructed differently; researchers are mainly constructed as responsible initiators and caretakers, while users are constructed as powerless and vulnerable	NA	NA	The guidelines portray an unequal distribution of responsibility between researchers and users. Researchers are expected to lead the process, and their positions as the most powerful actors remain unchallenged. The described harmonizing, value-laden approach, resting on a (traditional) paternalistic discourse, may act to preserve the existing disparities in power between researchers and users described in the literature	The findings raise the question of whether the existing guidelines may function to reproduce dominant relationships within the collaboration process between users and researchers. Even though research guidelines are mainly portrayed in the literature as positive, it is important to recognize that different discursive formations of guidelines and policies exist, depending on the discourses that the actors draw upon, either consciously or subconsciously
Koren Solvang et al. (2021)	Discussion of dissimination process	NA	NA	User panel meetings	The service users engaged as co-researchers, based their contributions on their respective personal histories, represented an NGO and peers, applied their respective professional and educational backgrounds and, finally, engaged as concerned citizens	NA	NA	The findings add to the discussion of professionalization of user involvement by introducing a wider array of positions enacted than do the findings of previous studies. Researchers recruiting user panel members, as well as NGOs appointing candidates for user panels, are advised to consider a wide competence profile for possible candidates. A panel is also considered as a resource in confirming and elaborating on a study's findings	An added value was the professional backgrounds in health and educational services held by two user participants They could contribute to member checking of the analysis of professional work from their positions as service providers. Based on these characteristics of the panel discussions, projects appointing user panels could consider the possibility of more actively including panel discussions as part of the methodological design Another limitation concerns diversity in the panel. The members were predominantly white and middle class. Their homogenous social backgrounds and professional careers might have restricted the scope of inputs to the research process. However, some panel members had stopped working because of the consequences of their respective accidents and the panel discussions often contained reflections on the interests of accident-injured people in more vulnerable positions than the panel members themselves were
Slåtsveen et al. (2021)	NA	NA	NA	Steering group meeting Workshop Brainstorming session Online survey	The votes for the top 10 research questions were evenly distributed, all rating above 40%, and the question voted in second place was selected as the main question for this doctoral degree project	NA	NA	The steering group members could have actively participated in the process of thematising, interpreting and developing the questions in the interim priority setting. Inviting some of the steering group members or another representative to be a coauthor of this article could also have contributed to richer and more nuanced perspectives and experiences of this NLR process. This would raise issues such as increased time consumption, the question of financially compensating the members, and finding members with sufficient time and interest to participate in such processes	Consideration should be given to identifying input from the service users separately if conducting a similar process in a larger project and involving other relevant representatives from different cultures and languages In projects with a higher budget and thereby possibilities to provide honorariums to those who use their spare time for this kind of work, honorarium must be considered. This is not only for acknowledging the time, resources and expertise given to the project but also to create a sense of equality among the members In a relatively small-scale project such as the present study, where time and resources are limited, it was not feasible to reach more service users or to run a larger campaign to attract them Critics have argued that, despite its democratic intentions, this way of employing user involvement does not necessarily empower patients, since the researcher retains – and may choose to wield – her power to define what a legitimate research question is and how to answer it
Sand et al. (2020)	NA	NA	NA	NA	NA	NA	NA	NA	When aiming at better use of PPI and thereby enhancing relevance and quality in health research, it is crucial to build a culture of mutual trust and a better understanding of the concept
Staats et al. (2020)	NA	NA	NA	NA	NA	NA	NA	After discussing comments and recommendations, we decided to implement most of the patient and informal caregiver responses in our research strategy Utilizing PAICPAIR as inspired by the INVOLVE guidelines has improved research quality through patient and informal caregiver inclusion, training, and support	NA
Stuhlfauth et al. (2020)	NA	NA	NA	Focus group	Participants stated they considered equity as a highly relevant and interesting topic	NA	NA	Users and researchers constructed equity in user involvement differently, but the difference was masked by an apparent agreement. Users and researchers drew on different storylines	The study revealed different perceptions about ‘equity in user involvement’ and implies that it is important to uncover and discuss these differences in collaboration processes
Stuhlfauth et al. (2019)	NA	NA	NA	Focus group	The focus group discussion revolved around different storylines that were portrayed in an intertwined and interdependent way. The different positioning of the two parties was related to different responsibilities, rights and duties in the research process. However, users and researchers were preoccupied with different aspects of the topics	NA	NA	The positions that users and researchers assume and ascribe throughout the process are constantly changing. Different positions in the form of dissimilar rights and duties create power differences and these stand out as barriers in the collaboration process. The different positions might challenge an equal collaboration between users and researchers and it seems that the ideal of coproducing research is hard to reach	The relationship between equity in user involvement in research and power needs to be studied further to understand how dilemmas, contradictions and paradoxes in the research process evolve
Mjøsund et al. (2018)	Abstract, poster, article, seminar production	NA	NA	Documents and texts produced while conducting the project, as well as transcripts from multistage focus group discussions with the research advisors, were analysed	The involvement of the research advisors varied throughout the different stages of the research process Features of the collaboration process were important for the research advisors to see themselves as valuable and competent contributors to the research process Six features are seen as guiding principles for a salutogenic service user involvement which promoted a structure and atmosphere facilitating research advisors to join the research team	NA	NA	Experiences from involving service users as research advisors in a mental health promotion project made us see the significance of the working environment A description of the Significant contributions from research advisors were promoted by facilitating the process of involvement. A supporting structure and atmosphere were consistent with a salutogenic service user involvement	Strength is the coauthorships with the persons involved as research advisors A team of research advisors can be a source of fun and energy, as well as enhance the research quality. Although the service user involvement in the project required extensive use of time to plan and manage the collaboration process. It may not be possible to overcome all barriers to service user involvement in all studies all of the time, but experiences might contribute to a more detailed understanding of how it can be achieved and thereby help improve the quality of service user involvement
Mjøsund et al. (2017)	One person from the advisory board was a handy helper in posters, presentations and article production	NA	NA	Analysis of data from interviews. Discussions were audiotaped, transcribed and interpreted following the guidelines for good qualitative analysis in interpretative phenomenological analysis studies	The advisory team became ‘the researcher’s helping hand’. Multiple perspectives influenced the qualitative analysis, which gave more insightful interpretations of nuances, complexity, richness or ambiguity in the interviewed participants’ accounts. The outcome of the service user involvement was increased breadth and depth in findings	NA	NA	Service user involvement improved the research quality in a nursing research project on mental health promotion. The interpretative element of interpretative phenomenological analysis was enhanced by the emergence of multiple perspectives in the qualitative analysis of the empirical data	Researchers using qualitative methodology should adapt service user involvement in health and nursing research projects •Nurses in clinical practice and service users should be aware of the synergy and power of multiple perspectives brought into decision-making in nursing and healthcare research and development
Natland et al. (2017)	NA	NA	NA	NA	NA	NA	NA	There is consensual agreement that the patient perspective helps supplement the perspectives of clinicians and researchers. Patient participation cannot be regarded as a mere alibi, but as equivalent in knowledge production to produce better health services through research. Here, we have emphasised that participation can be graded, and only the highest levels imply co-research. There must be room for a variety of approaches in research, and different stages of the project may be suitable for consultations as well as for collaboration (or even management)	An increased awareness of the many forms of user participation may help researchers see new opportunities for patient participation in the production of knowledge. If we explore the terrain, many opportunities may reveal themselves on the road from the ivory tower to the marketplace
Tangvald-Pedersen et al. (2017)	NA	NA	NA	Qualitative interviews/narratives	The tinkering approach created a space for the participant researcher and the academic researcher to follow their respective courses of inquiry, thereby adopting different stances within participatory research. The approach created space for transparency of the research process and dialogue about the intermediate and end results of the study	NA	NA	Tinkering user research participation advances beyond mere opportunistic eclecticism. Embarking on any research project guided by the principles of a wellordered science implies involvement and negotiations amongst those concerned regarding the distribution of the financial means, the setting of the research agenda and the use of the research results	
Moltu et al. (2013)	NA	NA	NA	Focus groups	Three core themes was identified that represent important coresearcher functions around which the participants developed a consensual understanding: the advocate for usefulness, the brakeman, and the interpreter	NA	NA	A practical implication of the categories we have found is that they equip researchers and service users with ideas for a potential mandate when collaborating to develop the coresearcher role, and suggest how this mandate can heighten the quality of the research process and outcome	There is increasing political will to involve service users in research, and some funding organizations expect such participation in research initiatives
Moltu et al. (2012)	NA	NA	NA	Focus group interviews	The themes were: self-definition, constructive differentiation and negotiations	Focus group	The dynamics of group engagement was subsequently confirmed as high by the participants. The group dynamics of negotiating to reach a consensual understanding were salient on these occasions. Hence, the findings selected for presentation and discussion here were experienced as important by the participants, and they represent instances where the group moved toward a shared understanding	As researchers funded by academic research organizations, the authors of this paper are expected to present findings in scientific journals such as this one. One challenge here is that the service user coresearchers in the project might not be accustomed to using the English language. Moreover, the particular form of language used in journal articles is quite dense. Both these issues risk lessening the availability of the findings for the participant coresearchers. The findings suggest that this involvement may be seen as a continuous and challenging process that involves negotiating one’s own role and mandate as a service user in relation to the academic world of research We are of the opinion that there may be added value in the collaboration between professionals and service users in research on mental health, but see the process of continually reflecting upon these issues as essential for actualizing the potential that lies within these approaches	It may be of relevance to both service user led and consultative studies in which defining oneself as a coresearcher, constructively differentiating oneself from other academics as well as negotiating ones loyalty and influence, as presented in our themes, can be seen as important challenges and tasks for the service user coresearchers
Kjeken et al. (2010)	NA	NA	NA	NA	NA	NA	NA	The surveys demonstrate that people with rheumatic diseases respond positively to participating in research and are highly competent at identifying important research issues. Participants had similar priorities for research The results indicate that willingness to participate varies, depending upon the aim of the study	Researchers should develop a patientfriendly study design, including routines to ensure that participants receive feedback on the study results Patients’ preferences for communication and dissemination of research also should be taken into account in future research projects Involving consumers as research partners is an effective way to enhance patient-centered research. To ensure good practice, existing principles and recommendations for successful consumer involvement should be used to guide researchers who are new to participatory research
*Sweden*									
Frögren et al. (2022)	NA	NA	NA	NA	Evaluation of participating in research in general	Lickert scale	Higher education is associated with with being actively involved in reasearch	NA	People with low education might not be as represented in PPI as people with low degree of education
Siira et al. (2022)	NA	NA	NA	NA	NA	NA	NA	NA	NA
Nyman et al. (2022)	NA	NA	NA	NA	NA	NA	NA	Participatory action research is an established method however the authors conclude that this method fail to explicitly demontrate the methodology	NA
Schandl et al. (2022)	NA	NA	Validated the study design	Questionnaire	Discussion of PPI and researcher prespectives of the PPI process	GRIPP	GRIPP was used for reporting PPI	PPI improves the relevance of the research and facilitated the dissemination of research findings	NA
Rudberg et al. (2021)	NA	NA	NA	The questionnaire was outlined and developed in accordance with earlier research in the area regarding patient involvement and adequate areas of research	The most prioritized areas of research were Balance and walking difficulties and Post-stroke fatigue	NA	NA	The potential to optimize life after stroke is vast and should be a frontier in stroke research. We found that the research areas most prioritized by the stroke patients differ with age and symptoms at stroke onset, indicating that rehabilitation strategies should be individualized and that this should be done already at discharge from hospital	NA
Berge et al. (2020)	NA	NA	NA	NA	Evaluation of being a PPI in an RCT	Interviews	Gives new perspectives and knowledge to to be shaped in the encounter and relationshipbetween the researcher and the frail older person	NA	NA
Kylén et al. (2020)	NA	NA	NA	A panel study will be implemented with different categories of knowledge users and researchers.A draft set of questions on attitudes and behavioral patterns related to research utilization and user involvement in research was compiled based on existing literature and input from the research team	NA	NA	NA	NA	The UserAge panel study will provide results that can be used to inform research funders and policy makers about the prerequisites needed to efficiently conduct research with user involvement. This can lead to more relevant findings to improve well-being in later life; improve the ability of research partnerships to benefit from diverse knowledge users’ local, lived, or applied knowledge; and jointly address the challenges of the aging society in the best possible way. Findings from the panel study may create conditions to improve approaches to involve knowledge users (eg, channels for recruitment, meet interests and expectations, handle barriers) to increase the quality and impact of research as well as give knowledge users participating in research a meaningful experience. In addition, knowledge derived from the panel study will contribute to the development of reliable and valid methodologies to evaluate research with user involvement
Warner et al. (2019)	NA	NA	NA	NA	Interviews from the case study	Survey and focus groups	Relatively positive findings with positive interactions and invitations to participate and low levels of of ideas being ignored	The findings indicate a need for thorough PPI preparation	NA
Iwarsson et al. (2019)	NA	NA	NA	Integrated in the management, a User Board and an External Advisory Committee composed of researchers and users give important input and monitor the overall development of the program	NA	NA	NA	NA	UserAge program is an example of a major research endeavor with potential to inform research with and about user involvement in research on aging and health. Taken together and communicated in the international scientific community as well as in a wide range of public and policy arenas, the empirical results, capacity-building, and modeling efforts will have an impact not only on the present situation but also on the future
Acosta et al. (2019)	NA	NA	NA	Online survey questionnaire Workshop	Together with research priorities retrieved from five different current guidelines, 94 uncertainties were expressed A shortlist of 24 uncertainties remained after processing for the final workshop. After the priority-setting process, using facilitated group format technique, the ranked final top 10 research uncertainties were listed	NA	NA	These ranked top 10 important research priorities may be used to justify specific research in aortic dissection and to inform healthcare research funding decisions	Patient involvement resulted in a more effective research agenda regarding AD for better healthcare than if research uncertainties would have been prioritized by physicians and other caregivers alone The findingsare strengthened by the transparent joint JLA process involving both patients and caregivers. One limitation was the possibility of subjective opinions and experiences expressed by the steering committee members, which might have affected processing and prioritization
Kylén et al. (2022)	NA	NA	NA	NA	NA	Survey	Different attitudes towards user involvement between the older population and the researchers	NA	NA
Kumlien et al. (2022)	NA	NA	NA	NA	NA	NA	NA	This prority setiing partnership study finds a top ten list of research priorities that reflect a swedish context, but might not be appropriate for other countries	NA
Malm et al. (2022)	NA	NA	Evaluated by researchers	Interviews	Discussion about a rationale for for a more limited involvement as too extensive involvement could hinder some people to participate	NA	NA	Involving people in too complex research studies might be too challenging for the participants	NA
Malm et al. (2019)	NA	NA	NA	Individual interviews	Core findings included carers’ discussions of the perceived challenges and benefits of their involvement in research, both generally and more specifically, in the context of their involvement in the development of a national carer strategy	NA	NA	Highlights the fact that involving carers in R&D work is complex and possesses several more unique aspects than user involvement in general, arising from carers’ basis in a family context and the carer identity. Due to varying prerequisites for involvement and differing needs for support, it is not feasible to have a general recipe describing how carer involvement can be realized in practice. Genuine carer involvement in research and policymaking demands a high level of engagement from all involved, otherwise there is a risk of carer involvement becoming tokenistic	Future research should seek the voices of carers who are not organized or who may be hard to reach, as well as explore the reasons why more female than male carers participate in R&D work, and/or further develop the CRAC framework Limitations included the relative lack of male carer participants and the convenience sample
Bergsten et al. (2014)	NA	NA	NA	Focus groups	The inventory of research ideas and areas of importance from the patient perspective resulted in several aspects of living of a chronic disease being highlighted, mostly focused on the patient’s dignity, identity and quality of life An overview of research ideas and areas of importance from the patient perspective	NA	NA	NA	The involvement of patients could help researchers to stay in tune with patients’ needs.The involvement of the people who are affected by the diseases in the planning of their healthcare, as well asin the field of research, could be a way to develop healthcare in chronic diseases
Carlsson et al. (2006)	NA	NA	NA	Open ended questions were mailed to participants	The responses to the question revealed four themes: the impact of processes that occur within the network, the impact of learning, the impact of innovation and development in cancer care, and the impact of PACP members’ personal cancer experience	NA	NA	The participants reported that relationships were formed and personal development occurred within the network. The major theme in both groups was the impact of processes that occurred within the network, but the PACP members also reported a greater knowledge and a decrease in feelings about possibilities of influencing health care	Networks and collaborations between PACPs and the health care sector are valuable, although PACP members and HCPs perceive them differently
*Finland*									
Jones et al. (2020)	NA	NA	NA	Interviews	NA	NA	NA	The stories that PPI experts tells are accounts to contruct to serve specific purposes	NA
Jones et al. (2017)	NA	NA	NA	NA	NA	NA	Health policy documents often equate involvement with choice making by service users and customers; or as involvement in service development by experts. In both of these cases, involvement is depicted as an individual activity that requires personal responsibility and specialist knowledge. Although involvement opportunities have overall increased, they are primarily available to people that are “participation ready” and able to adopt roles promoted in policies	Health policy documents produce one interpretation of involvement, nevertheless it is important that diverse groups of patients, the public and health professionals participate in the discussion and express their views, which may differ from those of policy makers	

NA = Not Available due to missing reporting or not applicable to report

**Table 3:** Characteristics of sources (n = 56) from the gray literature search based on origin and institutions

Institution	Type of document*	Purpose	Findings and/or conclusion regarding PPI	Target group	References
*Denmark*					
**Ministries** Ministry of Health	Announcement of application of pool	The pool is targeted gathering knowledge about methods, implementation and dissemination of initiatives that strengthen patient and relative involvement	NA	Healthcare professionals and managements in regions, municipalities, patient associations, knowledge centers etc. as well as citizens and patients	(1)
Ministry of Education and Research	Catalog of the National research strategic announcement	The FORSK2025 process must provide a consolidated overview of the most important research areas of the future. It will provide a basis for the political prioritization of strategic investments in research It is the hope that the catalog will also serve as inspiration in the work of prioritizing research funding and / or strategic focus areas on e.g. universities, artistic educational institutions, MVUs, business academies, GTS institutions and in public and private funds	The catalog is the result of a comprehensive mapping and dialogue process An essential path to achieve socially value creation is through research collaborations where disciplinary boundaries are being crossed, and users involved In this context, it is important that the research focuses on the users' research needs, which increases the likelihood that new knowledge quickly finds solid use in the business and the public sector	Business community, organizations, municipalities, regions and ministries, knowledge institutions and a wide range of other stakeholders	(2)
**Universities** University of Southern Denmark	Ph.D. course	Patient and public involvement (PPI) in Research and previous PhD course in PPI (M Hørder co-organizer) The aim of this course is to introduce the concept of PPI and take an analytical and critical view on the processes and potential outcomes of PPI. The course will focus on the various kinds of barriers that the researcher meets when she/he decides to involve patient representatives in research projects. There will be a special focus on the role of researchers, on the role of patient representatives and the interaction of patients and researchers that constitutes PPI	NA	Ph.D. students enrolled in the Faculty of Health Sciences at SDU, as well as employees at the Faculty of Health sciences at the University of Southern Denmark	(3)
University of Southern Denmark	Lecture	Experiences and considerations regarding boundaries for meaningful patient involvement in research	NA	NA	(4)
University of Southern Denmark and Knowledge Center for User Involvement (VIBIS)	Report	The patient as a partner in Danish health research: Knowledge sharing and mapping of patient involvement in research in Denmark	A crucial barrier is to translate the strategic initiatives into visible traces in the concrete research projects. There is a need for managerial and other support for researchers and research leaders. First and foremost, through learning that can happen in many ways, but most successfully by establishing collaborations and networks with other researchers at home and abroad It has therefore also been the intention of the knowledge sharing project to establish lasting network collaboration with the dialogue partners we have met at the strategic level (research institutions, foundations, etc.) and at the project level. There have generally been positive indications of wanting to join such a forward-looking network collaboration Although implementation has been initiated and promoted—e.g. through the knowledge sharing project—it will be crucial over the coming years to anchor processes more broadly and in a more structured way, as well as not least to coordinate and evaluate the efforts To ensure such continued implementation, coordination and evaluation, a "National Network for Patient Involvement in Health Research" will be the right tool	Researchers	(5)
University of Southern Denmark	Article	Information on the involvement of patients in research	The prevalence is increasing. A story about PPI	Researchers and health professionals	(6)
University of Southern Denmark	Article	Patients as partners in research: The challenges for researchers of patient involvement	NA	NA	(7)
University of Southern Denmark	Article	The researcher's perspectives on patient involvement in research	NA	NA	(8)
University of Southern Denmark	Webpage	To improve the health and well-being of patients and relatives and their experience with the health system by conducting health research in collaboration with patients and relatives	NA	Healthcare researchers	(9)
Aalborg University	Workshop	Opportunity to gain insight into and discuss experienced Danish senior researchers' experiences with user / patient involvement	NA	NA	(10)
Aalborg University	Discussion paper	Discusses how citizens’ social position may matter for co-creation in health research by drawing on relevant research literature	Focuses on the risk in patient and public involvement of reproducing health disparities through co-creation of knowledge	Healthcare researchers	(11)
Aarhus University	Webpage	Providing a forum for people from different organizations to collaborate on patient involvement intervention research, training and implementation ResCenPI's activities relating to involving patients as partners in research: 25 + researchers are conducting research with patient partners	NA	People from all health-related stakeholder groups	(12)
University of Copenhagen	Debate	Patients must be involved in medical research	NA	NA	(13)
University of Copenhagen	Ph.D. course	To introduce the concept of PPI. Teachers included both researchers and patients	NA	Researchers	(14)
**Patient organizations and funding bodies** Danish Patients	Arrangement	Can patients and relatives be involved in the research process? What can it contribute? And what significance does it have for the research process? The focus is on the "patient's premises"	NA	Danish patients	(15)
Danish Patients	Debate	But it is extremely important that the strategy is not “only" a growth strategy, because then we risk that the potential is not realized, and on the contrary leads to a development that does not benefit the patient and the citizen. The core of the strategy should be to create value for the patient. We must ensure that patients and citizens actually benefit from the development. The strategy should therefore have a strong focus on the involvement of patients and citizens—and of course health professionals, who together with the patient and the citizen must use the technologies	Involvement of patients and healthcare professionals is necessary to create value	Danish patients	(16)
The Danish Cancer Society	Policy	To involve patients in the research process	Involving patients in decision-making processes about research prioritization and scientific assessment of research helps to ensure that the research focuses on the topics and issues that are most important to Denmark and the cancer patients—and not only on the topics and issues that only seem interesting from the researchers' perspective. The Danish Cancer Society has therefore decided that cancer patients must always participate in scientific assessment committees	Cancer patients in Denmark	(17)
Research Panel	Information	The research panel is a Danish voluntary patient community that aims to promote research and development of new treatment in Denmark through participation in clinical research	The research panel is a Danish voluntary patient community that aims to promote research and development of new treatment in Denmark through participation in clinical research	Patients in Denmark	(18)
The Danish Cancer Society	Information	Research, Annual report 2021	In 2020, the Danish Cancer Society's Center for Cancer Research got its own panel of patients and relatives. It brings patients and relatives closer to the research and incorporate their perspective, experiences and input into concrete research projects The panel consists of nine patients and relatives, who meet twice a year with researchers who presents them with concrete research projects and asks a series of questions	Politicians and contributors as well as researchers	(19)
Danish Heart Foundation	Recommendation/ value set	Practical recommendations, including processing of applications, purpose of user involvement, where in the research process users can be involved and recruitment of users	User involvement is an assessment criterion in applications for support. There must be a plan for user involvement in the specific research project or an explanation of why user involvement is not an option or is not relevant in the project in question The purpose of user involvement in health research is among others to increase the relevance and value of research results for users and to increase the societal impact of research	Researchers (applicants)	(20)
Novo Nordisk Foundation	Recommendation/ set of values	NA	NA	Researchers	(21)
Trygfonden	Set of values	Research strategy	The research challenge in patient involvement is twofold. First, models for user involvement that can be developed and tested integrated into daily practice, and which makes immediate sense to both patients and therapists. Second, there is a need to develop a better understanding of the areas in which comprehensive patient involvement can raise the quality of a treatment	Researchers	(22)
The Danish Kidney Association´s research fund	Policy	NA	Criteria: The research must be initiated by or in collaboration with kidney patients. The research must involve patients	NA	(23)
Velux Foundations	Recommendation/ set of values	NA	Research with user involvement can be ensured, for example, by users being included as co-researchers, as part of the steering group, or in panel participation, all of which continuously contribute with relevant perspectives on the research work. They can take an active part in part or all the research, from the formulation of the project idea to the formulation of the conclusions, and even contribute to the scientific dissemination	NA	(24)
**Research strategies for health research** Steno Diabetes Center	SDCC's Guide to User Involvement in Research Projects	A practical guide for researchers in how to involve users in the planning and development of research projects	A practical guide to involving patients in research	Researchers	(25)
Central Denmark Region/ Aarhus University Hospital	Regional strategy for research in the field of health	Strategy to be realized in an action plan	Strategy to be realized in a regional action plan	Researchers	(26)
Region Zealand	Policy for health research 2019–2022 Guiding visions for the regional council strategy	Vision to develop a healthcare system on patients' terms by involving patients and relatives in a strong partnership	Goals are; Regional research councils are attached to the patient and relatives' representative, Research funds are awarded with requirements for patient involvement, Models are developed for best practices for patient involvement in research	Researchers	(27)
North Denmark Region /Aalborg University Hospital	Health research Strategy in the Region North Jutland 2014–2018	Strategy for research	At the same time, research organizations need to be strengthened with it purpose of establishing research collaborations in interaction easily between hospitals, general practice, the municipalities and the patients themselves	Researchers and health professionals	(28)
The Capital Region of Denmark	Document	Strategy for research	To prioritize and strengthening patient involvement in research—both in identifying research questions and in following up on the perceived effect and value of treatment and trajectory	NA	(29)
Hvidovre Hospital	Strategy for research	Strategy for research	Focus area on patient involvement	Researchers and health professionals	(30)
Norway					
**Ministeries** The regional health care institution Helse-Sør Øst RHF	Announcement of application of pool	A plan for user participation must be included as an element in the project description and will be part of the application assessment. The project description must explain the extent to which users are involved in the planning and implementation of the project, or why this is not appropriate	NA	Researchers	(31)
National program for clinical treatment research (owned by the four regional health care institutions; Helse Sør-Øst, Helse Vest, Helse Nord and Helse Midt-Norge)	Report	The program seeks to safeguard the user perspective in research, and user participation is therefore made mandatory for all projects. The the overall goals for user participation in clinical studies are to contribute to increased utility, better quality and greater relevance for the health service	All projects which are allocated funds must facilitate the inclusion of patients from all four health regions. Since 2016, over NOK 850 million has been distributed to 48 large, national clinical treatment studies. The projects benefit Norwegian patients. opportunity to try out new and promising treatment. So far there are over 16,000. Norwegian patients have been included in clinical treatment studies in the program portfolio, and the inclusion of new patients is ongoing	NA	(32)
The regional health care institutions (RHF)	Report	Report about ongoing research and innovation activities in the specialist health care	Of the projects who have received regional funding, 61 percent of the research projects report that users have participated in the planning phase. The users have also participated in execution of projects, dissemination of results, through user panels and in steering or reference groups For research projects 60 per cent of users come from user and patient organizations	Healthcare professionals and managements in state, regions, municipalities, patient associations, knowledge centers etc. as well as citizens and patients	(33)
**Universities** University of Bergen	Ph.D. course description	The course is designed to facilitate patient and public involvement in medical and health research	Spread knowledge regarding PPI within research Being able to assess and convey the added value of patient and public involvement and initiate productive user involvement in own research projects	The course is open to researchers, postdocs, students (PhD, master, students in the Medical Student Research Program) and others who are interested	(34)
University of Bergen	Course	The main objective of the course is to develop the participants’ capacity to assess and convey the value of patient and public involvement in general, as well as promoting productive user involvement in participants’ research projects	The course aims at creating a platform for competence development and networking across professional- and user roles, facilitating communication and sharing of experience from multiple perspectives	The course is a collaboration between Neuro-SysMed and the Centre for Cancer Biomarkers CCBIO, initiated by Neuro-SysMed in line with their strong focus on patient participation In addition, 18 patient representatives attended the course	(35)
University of Oslo	Webpage	To promote patient involvement in rehabilitation related research in Norway	The project involves a systematic gathering of information from service users regarding their experiences and expectations, which will form the basis of the development of a guide that can be utilized to facilitate patient involvement in research	Researchers	(36)
University of Stavanger	Report	The aim of this strategy is that patient and stakeholder involvement (PSI) becomes an integral feature across all stages of the research process in all SHARE affiliated projects, from prioritization and planning, management, and conduct, to the dissemination of findings and implementation of change based on results	Genuine patient and stakeholder involvement can improve research quality and the relevance of research themes and outcomes	Researchers and PhD students at SHARE research projects, including PhD projects. Guidance, organizational policies and training on PSI for researchers will be developed	(37)
NTNU – Norges Teknisk-Naturvitenskapelige Universitet	Webpage	The HUNT Study is a longitudinal population health study in Norway. They write that: We are completely dependent on HUNT participants' experiences and input to uncover needs, solutions, and challenges. That is why participants are involved in all our investigations, from the planning stage to the final evaluation	NA	Researchers and service users	(38)
NTNU	Webpage	The research group for patient education and user participation (PEP group, from Patient Education and Participation), researches the perspective of patients/users in the health service and what promotes a patient-centered and learning health service. We are a broadly composed research group with participants from various fields and institutes	NA	Researchers and service users	(39)
University of Tromsø	Webpage	Young people with mental health challenges participate in research. The aim is to improve the services they themselves need	In the project SunRise, young people from Goza and Tromsø are included in all project phases. The young people have been involved from the very beginning when the research applications were designed, to reference groups along the way. At the end, when the project is to be evaluated, they also get involved	Researchers and service users	(40)
Oslo Metropolitan University	Webpage	The bridge-building initiative at the Faculty of Health Sciences aims to link research, education, and clinical practice more closely together. By identifying the needs of the users, we must ensure that research and education are relevant and beneficial to society. Need-leed research must answer questions that have not already been answered through previous research (knowledge gaps) and whose relevance has been legitimized and prioritized by patients, relatives and/or healthcare personnel. The process will also identify so-called "unknown knowns", i.e. questions that have been answered through previous research, and which represent knowledge that can be transferred to users and fields of practice	The bridge-building initiative comprises several research projects, all of which are based on the principles of needs-identified research	Researchers and service users	(41)
VID vitenskapelige høgskole, Fakultet for helsefag Høgskolen i Innlandet. Fakultet for helse- og sosialvitenskap, OsloMet – storbyuniversitetet, Fakultet for helsevitenskap	Book	The purpose of the book is to reflect critically on user participation in research	The anthology has its origins in the authorities' increasing expectations and demands for user participation as part of the work to democratize and increase the utility of health and welfare research The main part of the book's chapters is based on concrete projects where co-production of knowledge between researchers and other actors has been central	Researchers, PhD, and master's students, but also user organizations and services that participate in health and welfare research	(42)
Høgskolen i Innlandet. Fakultet for helse- og sosialvitenskap	Webpage	Research group: Co-creation in research and service development. The research group consists of both researchers from the university and people with user experience. The research has particularly been aimed at people with mental disorders and the disabled	Several relevant research projects: such as: "Making user participation work", financed by the Norwegian research councils’ program for health- and care services	Researchers and service users	(43)
**Patient organizations and funding bodies** The research council	Portfolio plan for the health care sector	The portfolio plan is based on the government's long-term plan for research and higher education and The Research Council's strategy, and it is operationalized through investment plans and announcements. The portfolio plan basically applies in a 5–10-year perspective and was completed in 2022	User involvement and user participation are important priorities in the health portfolio and a requirement in many announcements. Users of health and welfare research ranges from politicians, health authorities, the health, care and welfare services, including healthcare personnel, and the healthcare industry for residents (citizens), patients and patients and user organizations	Government and researchers	(44)
The National Association for Public Health	Action plan for the National Association for Public Health (2022–2026)	Aim to contribute to user participation in research in both dementia, heart and vascular disease and wider public health research	NA	NA	(45)
**Research strategies for health research** Regional health enterprises	A guide to User Participation in Research Projects	A practical guide for researchers in how to involve users in the planning and development of research projects	A practical guide to involving patients in research within the specialist care services	Researchers and service users	(46)
The cancer union and the Dam foundation	Guide	A guide for researchers on how you can include users in research projects	A practical guide to involving patients in research	Researchers and service users	(47)
*Sweden*					
**Ministeries** The National Board of Health and Welfare	National action plan for increased patient safety in Swedish health care 2020–2024	The Action Plan aims to strengthen the staunch work of the principals in the systematic patient safety work and to help prevent patients from suffering from adverse events. The Action Plan shall also support and coordinate work on patient safety across the country	A chapter regarding the patient as co-creator	The Action Plan is developed in broad cooperation with authorities, representatives from principals and national organizations, representatives of private caregivers, patients and relatives, experts, and students and other stakeholders in patient safety	(48)
**Universities** University of Uppsala	Ph.D. course description	Patient and public involvement in research	The overall goal of the course is to build a basic understanding of PPI in health research. Further, the course will provide students with a simple toolkit to facilitate the application of the knowledge developed from the course in their own research	Ph.D. students enrolled at the University of Uppsala	(49)
University of Gothenburg	A description of the Centre for Person-centered care	The overarching objective is to support and carry out high quality research relating to person-centered care	On the webpage, they give some tips on how, when, and why patients and the public can be involved in research	Fellows, students and healthcare professionals	(50)
University of Gothenburg, Linné University and Högskolan Kristianstad, Lund University	A description of a research program	Within the UserAge programme (2017–2022), researchers study user participation to increase our understanding of the opportunities and challenges presented by the participation of knowledge users in research on ageing and health	The UserAge research program aims to increase understanding of how users can contribute at various stages in the research process	Researchers, politicians, business, public sector, interest organizations and private individuals	(51)
**Patient organizations and funding bodies** Forte, research council	Report	A short report about user participation –Research with and about user participation and the meaning of inclusive research Forte is a research council that finances and initiates research to support people's health, working life and welfare	It is complex and complicated to evaluate the effects of user participation in research (Barber et al., 2011) and consequently such studies are unusual. To that extent evaluation efforts are reported, they are usually based on "anecdotal evidence" (Fudge, Wolfe & McKevitt, 2007). In a recently published literature review limited to user participation in health and social services, with adults as the target group, a larger number of articles is where the effects of research with user participation were reported in different ways (Brett et al., 2014)	Researchers	(52)
Svenska Läkaresällskapets	Webpage	The need for user participation must be considered and discussed and, where appropriate, described in SLS project applications	NA	Researchers and service users	(53)
*Finland*					
**Ministeries** Ministry of Education, Science and Culture	Webpage	Encouraging researchers to seek bold, new research initiatives that can solve health issues related to major public health diseases	Many agencies funding health research request researchers to consider patient and customer perspectives already when planning their research	Researchers	(54)
*Iceland*					
**Universities** University of Iceland	Webpage	The University of Iceland advertises new grants to support academic staff in public outreach and active participation in society based on their research and specialist knowledge	NA	Academic staff	(55)
**Patient organizations and funding bodies** The Icelandic Cancer Society	Webpage	Overall description /homepage	NA	NA	(56)

^*^(e.g. research paper/notation/ information report/ recommendation/ policy/strategy/guidance/set of values/ debate post/letter to the editor)

NA = Not Available

(1) Authority DH. Puljeopslag: Vidensopsamling om metoder, implementering og udbredelse af indsatser, der understøtter øget patientinddragelse i sundhedsvæsenet. Copenhagen: Sundhedsminiteriet; 2016

(2) Science DAfHEa. FORSK2025 – fremtidens løfterige forskningsområder. Copenhagen: Uddannelses- og Forskningsministeriet; 2017

(3) Faculty of Health Sciences UoSD. Patient and public involvement i research Odense: University of Southern Denmark; 2018. Available from: https://www.sdu.dk/en/forskning/phd/phd_skoler/phdskolensundhedsvidenskab/phd_courses/course_archive

(4) Thuesen J, editor Erfaringer og overvejelser vedrørende grænser for meningsfuld inddragelse af patienter i forskning. Grænser for inddragelse: Seminar i Enhed for Brugerperspektiver; 2018; Odense: University of Southern Denmark

(5) Sandvei M, Hørder M. PATIENTEN SOM PARTNER I DANSK SUNDHEDSFORSKING. Vidensdeling og kortlægning af patientinddragelse i forskning i Danmark. Odense: Forskningsenheden for Brugerperspektiver, Institut for Sundhedstjenesteforskning; 2018

(6) Jørgensen BMK. Patienter skal inddrages i medicinsk forskning Odense: University of Southern Denmark; 2017. Available from: https://www.sdu.dk/da/nyheder/nyhedsarkiv/nyheder/arkiv_2017/november/medicinsk+forskning

(7) Nielsen MK, Hørder M. Patients as partners in research – The challenges for researchers of patient involvement. Research OUTREACH. 2020;117

(8) Nielsen MK, Sandvei M, Hørder M. Forskerens perspektiver på patientinddragelse i forskning. Ugeskr Læger. 2018;180

(9) HOSPITAL OU. Center for Forskning Sammen med Patienter og Pårørende: ODENSE UNIVERSITY HOSPITAL; 2023. Available from: https://ouh.dk/forsa-p

(10) Petersen KS, Staley K, Overgaard C, Høstgaard AMB. Workshop on PATIENT INVOLVEMENT IN RESEARCH Aalborg: Aalborg University; 2018. Available from: https://vbn.aau.dk/da/activities/workshop-on-patient-involvement-in-research

(11) Stage JT. Business as usual? Inequalities in patient and public involvement in health research. Academic Quarter | Akademisk kvarter. 2022(24):71–85

(12) Health DoP. Research Centre for Patient Involvement -ResCenPI Aarhus: Aarhus University; 2022. Available from: https://ph.au.dk/rescenpi

(13) Kristensen DB, Bruun B, Lindskov M, Brorholt G. Debat: Patienter skal inddrages i medicinsk forskning. Altingetdk. 2017

(14) Sciences FoHaM. Patient and public involvement in health research Copenhagen: University of Copenhagen; 2023. Available from: ttps://phdcourses.ku.dk/DetailKursus.aspx?id=110637&sitepath=SUND

(15) Patients D. Involvering af patienterne i forskning Copenhagen: Danish Patients; 2018. Available from: https://danskepatienter.dk/arrangementer/involvering-af-patienterne-i-forskning

(16) Patients D. Sæt patienterne i fokus i ny life science-strategi Copenhagen: Danske Patienter; 2020. Available from: https://danskepatienter.dk/politik-presse/nyheder/saet-patienterne-i-fokus-i-ny-life-science-strategi

(17) Society TDC. KRÆFTENS BEKÆMPELSES FORSKNINGSPOLITIK Copenhagen: The Danish Cancer Society; 2018. Available from: https://www.cancer.dk/dyn/resources/File/file/9/9269/1620740529/forskningspolitik.pdf

(18) Forskningspanelet. Velkommen til Forskningspanelet Copenhagen: Forskningspanelet; 2022. Available from: https://forskningspanelet.dk/

(19) Society TDC. Kræftens Bekæmpelse Forskning 2020 FORSKNINGSÅRSRAPPORT Copenhagen: The Danish Cancer Society; 2020. Available from: https://www.cancer.dk/dyn/resources/File/file/3/9113/1611910143/forskningsaarssrapport-2020.pdf

(20) Foundation DH. Brugerinddragelse i forskning Copenhagen: Hjerteforeningen; 2022. Available from: https://hjerteforeningen.dk/stoettetilforskning/kriterier-for-bedoemmelse/brugerinddragelse/

(21) Foundation NN. Projektstøtte til eksplorative studier i sammenhængende behandlingsforløb Copenhagen: Novo Nordisk Fonden; 2021. Available from: https://novonordiskfonden.dk/grant/projektstoette-til-eksplorative-studier-i-sammenhaengende-behandlingsforloeb/

(22) TrygFonden. TrygFondens forskningsstrategi Virum: TrygFonden; 2014. Available from: https://www.tryghed.dk/viden/publikationer/trygfondens-forskningsstrategi

(23) fund TDKAsr. Støtte til Forskning inden for nyrelidelser Copenhagen: The Danish Kidney Association's research fund; 2022. Available from: https://www.legatbogen.dk/nyreforeningens-forskningsfond/stoetteomraade/6268

(24) Foundations V. Brugerinddragelse i forskning Copenhagen: Velux Foundations; 2022. Available from: https://veluxfoundations.dk/da/brugerinddragelse-i-forskning

(25) Copenhagen SDC. SDCCs Vejledning i Brugerinvolvering i Forskningsprojekter Copenhagen: Steno Diabetes Center Copenhagen 2020. Available from: https://www.sdcc.dk/presse-og-nyheder/nyheder/Documents/SDCCs%20Vejledning%20i%20Brugerinvolvering%20i%20Forskningsprojekter.pdf

(26) Region CD. Region Midtjyllands strategi for forskning på sundhedsområdet Viborg: Region Midtjylland; 2020. Available from: https://www.rm.dk/siteassets/sundhed/region-midtjyllands-strategi-for-forskning-pa-sundhedsomradet_19februar2020.pdf

(27) Zealand R. Forskning på forkant

Region Sjællands politik for sundhedsforskning 2019–2022 Sorø: Region Zealand; 2019. Available from: http://publikationer.regionsjaelland.dk/data-og-udviklingsstoette/forskning-paa-forkant/?page=24

(28) Region TND. EN SUNDHEDSFORSKNINGSSTRATEGI SOM UDGANGSPUNKT FOR BEDRE SUNDHED OG BEDRE VELFÆRD: The North Denmark Region; 2013. Available from: https://rn.dk/~/media/rn_dk/sundhed/til%20sundhedsfaglige%20og%20samarbejdspartnere/forskning/forskning%20og%20analyser%20i%20sundhed/sundhedsforskningsstrategigodkendtp%c3%a5regionsr%c3%a5detsm%c3%b8de.ashx

(29) Hovedstaden R. Forskningsstrategi. Kliniknær forskning som fundament for et evidensbaseret sundhedsvæsen 2023. Available from: https://www.regionh.dk/presse-og-nyt/pressemeddelelser-og-nyheder/PublishingImages/Sider/Patienten-skal-i-centrum-for-regionens-forskning/Forskningsstrategi%20for%20Region%20Hovedstaden%202023.pdf

(30) Hospital H. Forskningsstrategi—Det handler om liv Hvidovre: Hvidovre Hospital; 2022. Available from: https://www.hvidovrehospital.dk/forskning/om/Sider/forskningsstrategi.aspx

(31) RHF HS-Ø. Utlysning av regionale forskningsmidler for 2023 2022. Available from: https://helse-sorost.no/helsefaglig/forskning/forskningsmidler/utlysning-av-regionale-forskningsmidler

(32) research Npfct. Årsrapport 2021. Available from: https://kliniskforskning.rhf-forsk.org/wp-content/uploads/sites/2/2022/10/Klinbeforsk_rapport_2022_online_compress.pdf

(33) helseforetak) Rher. Nasjonal rapport om forskning og innovasjon fra spesialisthelsetjenesten 2021. Available from: https://helse-vest.no/seksjon/planar-og-rapportar/Documents/Nasjonal%20forsknings-og%20innovasjonsrapport/Forskning%20og%20innovasjon%20til%20pasientens%20beste%202021.pdf

(34) Bergen Uo. Patient and public involvement in medical and health research Bergen: University of Bergen; 2022. Available from: https://www.uib.no/en/course/CCBIONEUR910

(35) VIDHAMMER ES, JEBSEN NL, SKÅR T. Bringing the patient perspective into research Bergen: University of Bergen; 2022. Available from: https://www.uib.no/en/k1/149883/bringing-patient-perspective-research

(36) Society IoHa. Patient involvement in Health Services Research Oslo: University of Oslo; 2022. Available from: https://www.med.uio.no/helsam/english/research/groups/patient-involvement-rehab-research/

(37) Healthcare SCfRi. Patient and Stakeholder Involvement. (PSI) Strategy (2020–2022): University of Stavanger; 2020. Available from: https://www.uis.no/sites/default/files/2020-08/SHARE%20PSI%20Strategy%202020-2022.pdf

(38) NTNU. Brukermedvirkning 2023. Available from: https://www.ntnu.no/hunt/brukermedvirkning

(39) NTNU. Patient Education and Participation (PEP) 2023. Available from: https://www.ntnu.edu/ism/pep#/view/publications

(40) universitet UiTNa. Forskning som inkluderer 2022. Available from: https://uit.no/nyheter/artikkel?p_document_id=794022

(41) University OM. Brobyggersatsingen. Available from: https://www.oslomet.no/om/hv/fou/brobyggersatsingen

(42) Sigrid Ø, Inger Marie L, Ole Petter A. Samproduksjon i forskning: Forskning med nye aktører. Place of publication not identified: Scandinavian University Press Universitetsforlaget; 2019

(43) sosialvitenskap HiIFfh-o. Samskaping i forskning og tjenesteutvikling. Available from: https://www.inn.no/forskning/var-forskning/samskaping-i-forskning-og-tjenesteutvikling/index.html

(44). Forskningsrådet. Porteføljeplan for Helse 2022. Available from: https://www.forskningsradet.no/portefoljer/helse/portefoljeplanen-for-helse/

(45). folkehelse Nf. Handlingsprogram 2022—2026. 2022

(46) helseforetak) Rher. Veileder for brukermedvirkning i helseforskning i spesialisthelsetjenesten 2018. Available from: https://helse-sorost.no/Documents/Forskning/Forskningsmidler/Maler/Utkast%20-%20veileder%20-%20endelig%20mai18.pdf

(47) Dam) TcuKatDfS. Håndbok. Brukermedvirkning i forskning. Available from: https://kreftforeningen.no/content/uploads/2023/03/handbok-brukermedvirkning-i-forskning.pdf

(48) WELFARE NBOHA. Act for safer healthcare: NATIONAL BOARD OF HEALTH AND WELFARE; 2020. Available from: https://www.socialstyrelsen.se/globalassets/sharepoint-dokument/artikelkatalog/ovrigt/2020-1-6564-english.pdf

(49) Uppsala Uo. Patient and public involvement in research Uppsala: University of Uppsala 2022. Available from: http://www2.medfarm.uu.se/utbildning/forskarniva/for_doktorander/kurs897.html

(50) Care CfP-c. Patient and Public Involvement (PPI) in research – how, when and why Gothenburg: University of Gothenburg; 2022. Available from: https://www.gu.se/en/gpcc/patient-and-public-involvement-ppi-in-research-how-when-and-why

(51) University L. UserAge 2021. Available from: https://www.case.lu.se/forskning-vid-case/userage

(52) counsil FR. BRUKARMEDVERKAN. Forskning med och om brukarmedverkan 2015. Available from: https://forte.se/app/uploads/2015/05/fik-brukarmedverkan.pdf

(53) Läkaresällskapet S. Brukarmedverkan i forskningen. Available from: https://www.sls.se/vetenskap/sok-anslag/fonder/brukarmedverkan-i-forskningen/

(54) Finland Ao. Health from Science (TERVA) 2018–2022 Helsinki: Academy of Finland; 2018. Available from: https://www.aka.fi/en/research-funding/programmes-and-other-funding-schemes/academy-programmes/health-from-science-terva-20182020/

(55) Iceland Uo. The University of Iceland supports academic staff in public outreach Reykjavík: University of Iceland; 2020. Available from: https://english.hi.is/news/the_university_of_iceland_supports_academic_staff_in_public_outreach

(56) Society TIC. The Icelandic Cancer Society Reykjavík: Krabbameinsfélagið; 2020. Available from: https://www.krabb.is/english

### Characteristics of empirical and non-empirical papers

**Fig. 2:**
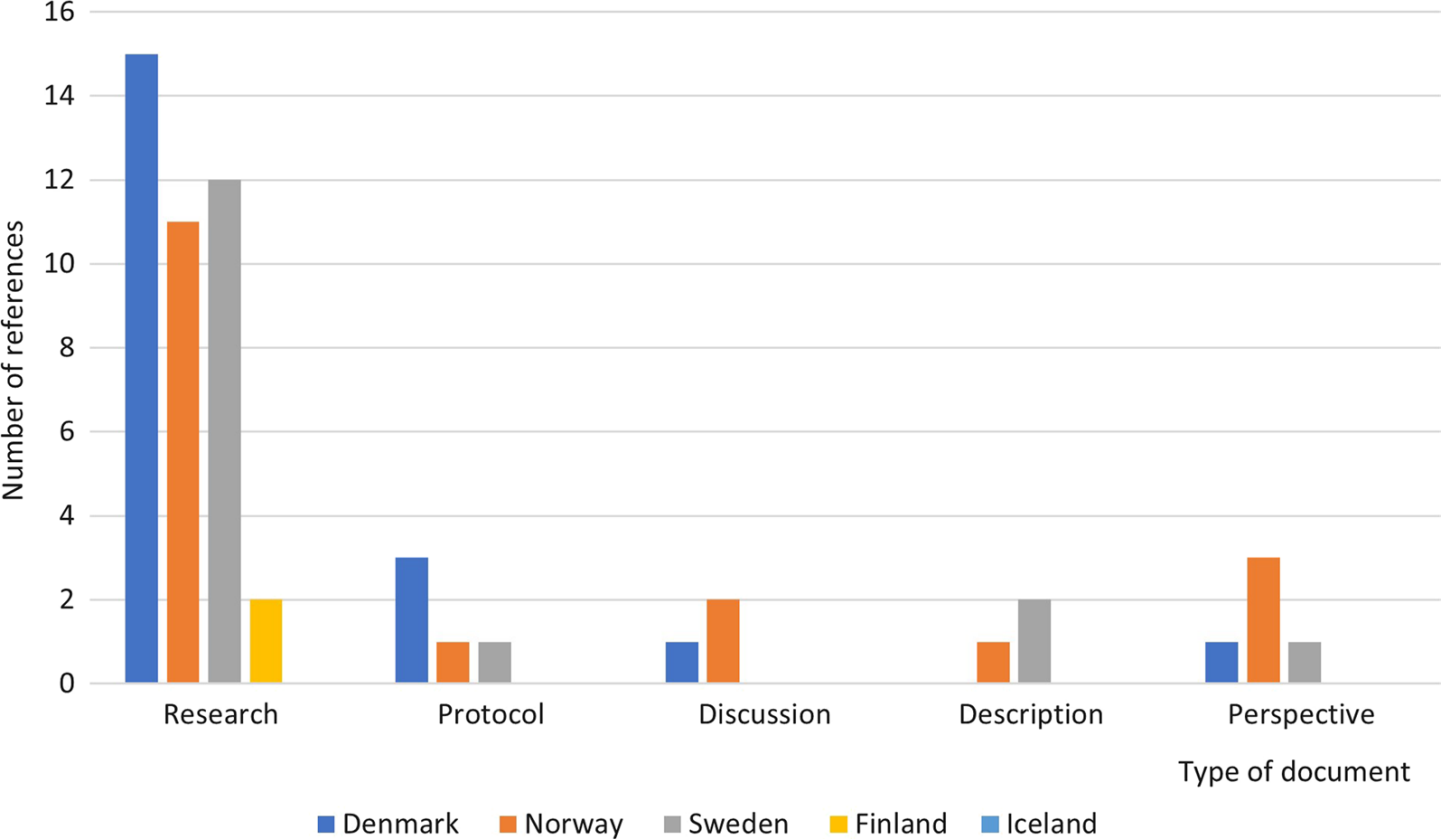
Distribution of the included papers (n = 56) in relation to categorization based on origin the categorization was performed in relation to how the authors of the papers explicitly defined their own paper

Thirty-nine papers (70%) were empirical studies that applied PPI in healthcare research and 17 (30%) were study protocols or non-empirical papers that reported on aspects of PPI. Papers were published in 2006–2023, and first authors were affiliated with Denmark (n = 20), Norway (n = 18), Sweden (n = 16), Finland (n = 2), and Iceland (n = 0). Distribution of the included papers in relation to type of document is presented in Fig. 2.

Among the 39 empirical papers, 26 (67%) were qualitative [15, 24–48], four were case studies [49–52], five quantitative [53–57], and four multi-methods studies [58–61]. The study populations from the empirical papers were patients with cancer (n = 8) [15, 27–29, 45, 51, 59], mental health illness (n = 8) [34, 35, 39, 42, 46, 49, 50, 61], cardiovascular disease (n = 2) [24, 54], kidney failure (n = 1) [52], traumatic brain injury (n = 1) [38], reproductive dysfunction (n = 1) [30], women with prior gestational diabetes mellitus (n = 1) [43], older adults and geriatric disorders (n = 7) [25, 26, 36, 40, 47, 56, 57], stroke (n = 2) [53, 55], ankylosing spondylitis (n = 1) [44], persons living with diabetes (n = 1) [60], and patients with rehabilitation needs (n = 2) [31, 48]. In four studies, no specific disease population was described [32, 37, 41, 58]. In 21 studies, solely patients were involved [27, 29, 30, 34, 35, 39, 40, 44, 45, 47, 48, 51, 52, 55–57, 61–65], six studies involved public [32, 36, 38, 42, 46, 58], 19 studies involved a combination of patients and public [15, 24–26, 28, 31, 41, 43, 49, 50, 53, 54, 59, 60, 66–70] and ten studies did not report whether they used patients, public or both [2, 33, 37, 71–77].

Of the 17 non-empirical papers, six were study protocols that utilized PPI methods, including a study with an exploratory and participatory design to improve resilience in healthcare [68], a qualitative study to identify and prioritize future cancer research agenda [67], a meta-epidemiological study investigating PPI in an intensive care setting [78], a quasi-experimental cohort research study on nurse-led consultations [64], a multiple method study to develop a core outcome set for intensive care unit patients [66] and a large-scale panel study evaluating PPI awareness in aging and healthcare research [70]. The remaining eleven non-empirical papers provided diverse perspectives on PPI [2, 62, 63, 65, 69, 71, 73–77]. Tangvald-Pedersen and Bongaardt offered an ideological perspective, describing participatory research in mental health within three ideologies, liberal, emancipatory, and caring, to balance demands of science and social relevance [76]. Beedholm and Frederiksen took an institutional perspective, discussing contextual and structural factors that impact use of PPI and introduces the concept of ‘patient logic’ [73]. Natland et al. examined PPI from an epistemological perspective, discussing how it affects knowledge production [75]. Kjeken et al., Sand et al., and Staats et al. provided methodological perspectives on PPI, describing research priorities, debating better ways to develop and evaluate PPI, and suggesting a framework for patient and informal caregiver participation in the research process, respectively [2, 63, 69]. Iwarsson et al. took a pragmatic viewpoint, describing potentials, problems, and challenges for health outcomes, concluding that capacity-building among elderly people and a specific model is beneficial for PPI [62]. Bergsten et al. shared a social and cultural perspective, describing how PPI in rheumatic diseases research planning sustained dignity, identity and quality of life for the researched group [65]. Bundgaard et al. highlight in a letter to the editor the need for debate of the utilization of PPI [71]. Siira et al. discussed the advantages and disadvantages of collecting information via online citizens and assess these panels potential in cardiovascular research [77]. Finally, Gilhus et al. discussed how PPI in Myastenia Gravis research could improve several aspects of research and clinical practice [74].

### Theoretical frameworks used to characterize PPI

Theoretical underpinnings of PPI were reported in Table 4 and included 25 papers. Twelve papers reported using a framework originating in the UK [27, 28, 41, 42, 51, 54, 55, 58, 60, 61, 69, 79]. One study used NIHR framework to engage patients at different phases of the research process [51], while Staats et al. used it as a project design and management guideline [69]. Other frameworks reported specific objectives for PPI, including positioning theory as applied by Stuhlfauth et al. and Koren Solvang et al., which elaborates on how people use language to situate themselves and others [31, 32, 38]. One study reported to use a cooperative inquiry described by Heron and Reason [42].

**Table 4:** Theoretical frameworks used to describe PPI in healthcare research in the Nordic countries

Framework/theory	Number	References
James Lind Alliance	5	Acosta et al. (2018), Piil et al. (2019), Slåtsveen et al. (2021), Solbakken et al. (2022), Kumlien et al. (2022)
The Dialogue model	1	Bergsten et al. (2014)
Three ideologies that advocate for user involvement and participant research described as liberal, market-based ideology, a survivor-led and emancipation-based ideology, and a healthcare and education-based ideology	1	Tangvald-Pedersen et al. (2017)
Positioning theory	3	Stuhlfauth et al. (2019), Stuhlfauth et al. (2020), Koren Solvang et al. (2021)
NIHR (INVOLVE)	4	Staats et al. (2020), Skovlund et al. (2020), Warner et al. (2021), Finderup et al. (2021)
Empowerment by Zimmerman and Rappaport	1	Jørgensen et al. (2018)
Cube Model by Gibson	1	Høeg et al. (2019)
The participatory approach	2	Kirk et al. (2021), Nyman (2022)
Health Canada Public Involvement Continuum	2	Finderup et al. (2021), Hansen et al. (2021)
UK Medical Research Council’s (MRC) framework	1	Thomsen et al. (2022)
A cooperative inquiry by Heron & Reason	1	Berring et al. (2021)
Patient and service user engagement in research by Shippee et al	1	Schandl et al. (2022)
Bammer’s stakeholder participation spectrum	1	Timm et al. (2022)
Patient‐Centered Outcomes Research Institute (PCORI)	1	Finderup et al. (2021)

### Research methods used to incorporate PPI and research stages

**Fig. 3:**
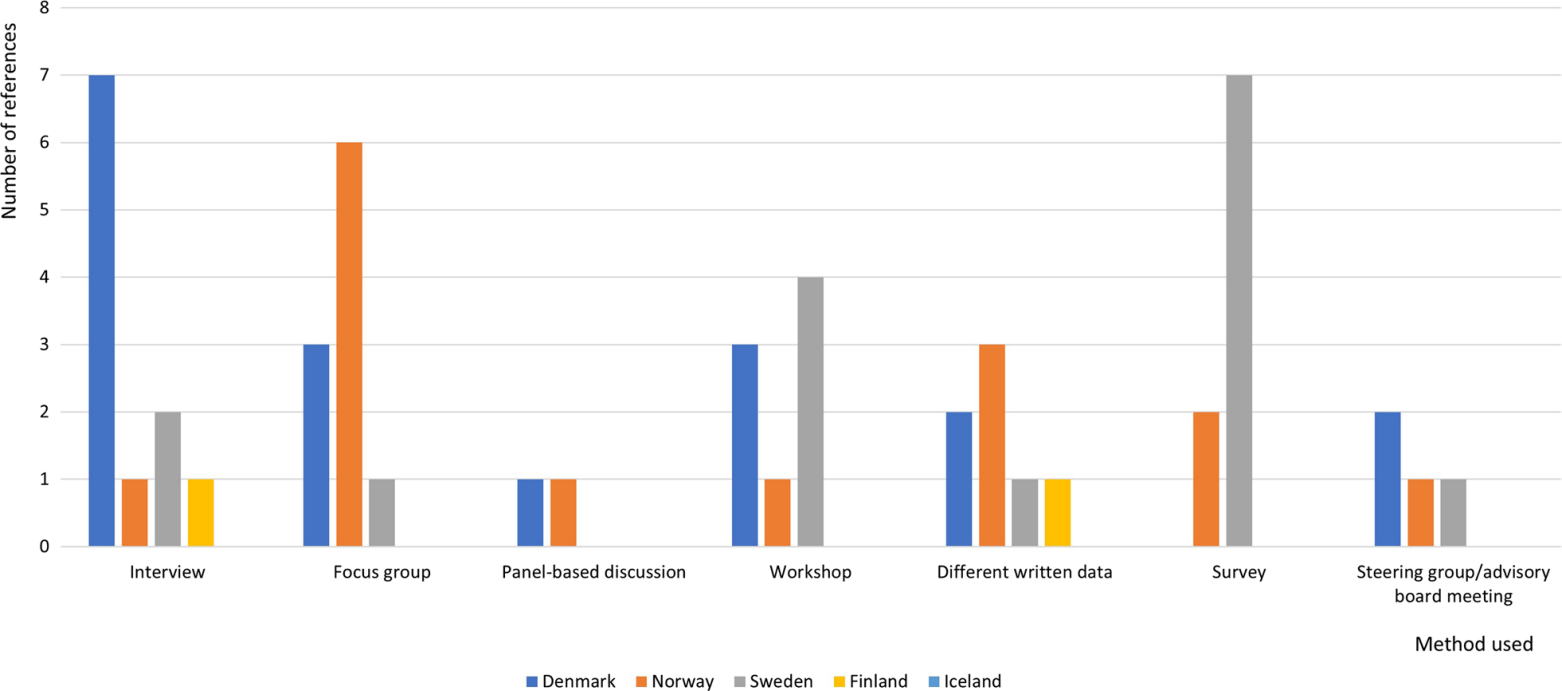
Overview of methods applied on origin. Thirty-two studies reported one or more methods; thus the number of methods is not related to the number of studies. Different written data covers e.g. sticky notes, email correspondence and transcripts from meetings

PPI was reported using diverse methods as visualized in Fig. 3. This included interviews [15, 24, 25, 27, 29, 36, 39, 41–43, 47, 52], focus groups [24, 27, 30–32, 34, 35, 40, 61, 65, 67], user panels or discussions [25, 38], workshops [29, 41, 48, 51, 54, 58, 60], written communications [15, 33, 49–51, 58, 59], surveys [53, 54, 58, 60, 61, 70] and steering groups or advisory boards [40, 44, 58, 62, 64].

Fourteen studies reported details of recruitment and sampling strategies for PPI, and included purposive sampling [34, 35, 44, 49, 50], convenience sampling [25, 55, 70], snowball sampling in three studies conducted by the same author [31–33], maximum variation [26], stratified sampling [62] and all possible sampling [24].

PPI was utilized across various stages of the research cycle with 19 studies reporting PPI to identify and prioritize research questions [25, 28–31, 40, 42, 44, 45, 48, 51–54, 58, 60, 61, 65, 67], and 15 studies reported employing PPI during the commissioning stage [28, 34, 36, 38, 40, 45, 50–52, 54, 58, 65, 67, 68, 70]. In the designing & managing stage, PPI was used in 17 studies [15, 26, 29, 38, 40–45, 48–52, 61, 64] with patients providing feedback on the interview guide, methods, procedures, and study planning. Ten studies reported to involved patients actively in research [29, 34, 35, 38, 44, 45, 50–52, 58], with one study reporting involving patients and family in analysis [50], and another reporting asking patients to help organize qualitative themes [35]. PPI was reported used in the dissemination process in five studies [38, 44, 49–51], including collaboration with patients on optimal methods for communication of research and including patients on advisory boards, in posters, presentations and publications [49, 50]. Two studies reported to use PPI in research implementation [51, 52], and six studies utilized PPI in research evaluation through interviews [29, 46], consultation on wording and expression [51], validating the study design [44, 45] and cancellation of a planned randomized controlled trial [30].

### Impact of PPI in research

Six papers reported and evaluated how PPI impacted the utilization of their research [29, 30, 44–46, 51]. The impact of PPI in the research differed in methods used for measuring impact and the specific study findings depending on the different approach to involve PPI. For example, Handberg et al. cancelled an RCT as PPI confirmed the irrelevance of the RCT [30]. Madsen et al. reported the enhancement and validation of their study by using PPI [44]. However, the specific impact of PPI was not outlined. Schandl et al. used GRIPP2 to report the use of PPI and found that PPI improved the relevance and facilitated the dissemination [45]. Nissen et al. concluded that PPI may contribute to enhancing the relevancy and quality of the research [15].

Through our gray literature search, we identified policies, practices, and initiatives of PPI in the Nordic countries from a range of sources, including government, academic institutions, funding bodies, patient organizations, and regional authorities to uncover the dissemination of PPI across different sectors. The evident diversity among these sources reflects the variations that exist across countries, institutions, and organizations within the Nordic region.

## Discussion

With the aim of exploring the current state, practice, and impact of PPI in healthcare research in the Nordic countries, our main finding was the intensified use of PPI in healthcare research in the selected geographic area. This was evidenced by an increasing number of publications and institutional initiatives in the recent years to involve patients and public in healthcare research. The most widely utilized theoretical frameworks were JLA and NIHR, both developed in the United Kingdom. This is not surprising, given the cultural and geographic proximity of the United Kingdom and the Nordic countries [80].

Although a universal definition of PPI might be lacking, we identified some studies in our scoping review that reported specific PPI aims in their research [27, 51]. A systematic review of cancer research in Europe found that PPI was mainly used in the first stages of research [8], which could be a sign of early days in the integration of PPI in the research process. This highlights the need for further exploration and development of PPI practices to ensure its full integration and meaningful impact on the research process.

Overall, this scoping review showed that PPI methods and approaches in Nordic literature is comparable to other international references [5, 8]. This suggests that the Nordic countries are aligning with global trends and recognizing the significance of involving patients and the public in healthcare research. In our scoping review, we found differences within the Nordic countries as Denmark, Norway, and Sweden contributed more references than Finland and Iceland. This can be explained by our language barriers and thus exclusion of documents in Finnish or Icelandic. Furthermore, Iceland has a smaller population size and may therefore contribute with fewer references. These findings are supported in a recent review focusing on PPI in Europe concluding that Scandinavian countries (Denmark, Norway, and Sweden) use PPI to a greater extent than Finland and Iceland [5].

The integration method of PPI in the research process exhibited variations across studies. We identified 14 empirical studies reporting the sampling strategy used in relation to PPI and found that the choice of strategy varied across studies. The absence of a sampling strategy could potentially lead to misrepresentation of involvement in the research process and affect the impact of PPI in relation to the study results [81]. Frögren et al. showed an over representation of people with higher education who were more willing to be involved in PPI [56]. This may pose a risk that groups with lower levels of education may not have been represented in PPI activities, potentially resulting in a biased representation. In our scoping review, we found a general absence in the level of detail provided regarding the comprehensiveness,scope, and timeliness of patients´experiences. Specifically, there is limited information about when these experiences occurred and the time since undergoing treatment. This could potentially impact the results of our study. Therefore, this review highlights the need for more detailed descriptions of PPI participants in future research to detect and address potential biases. To mitigate this risk, it is essential to actively strive for diversity and inclusivity in PPI initiatives. It is crucial to ensure that PPI strives for equal representation and prevent exclusion of those with limited resources to mitigate social inequality. The included papers in this scoping review did not prioritize sociodemographic descriptions of PPI participants. The references found in this review primarily involved patients or a combination of patients and the public. Only six studies solely involved the public. It may be relevant to investigate (in future studies) the distinctions between involving people with direct knowledge and experience of disease and treatment versus involving the broader public who have vested interest in ensuring the provision of high quality care.

In this review, interviews emerged as the preferred method for integrating PPI. However, the wide variety of qualitative and quantitative methods used in European studies involving different populations suggest that there is either a range of suitable research methods available or a potential lack of systematic approaches for PPI integration in the research process [8].

The most frequent type of reference identified in this review was empirical studies. In these studies, a discussion of aim and impact of PPI was rarely touched upon, perhaps due to word constraints in scientific  journals. As such, we still need more knowledge regarding the impact of PPI in healthcare research in the Nordic countries [5, 8, 9].

The references in our review did not sufficiently uncover the positive or negative impact of PPI in healthcare research. Empirical studies reported very little description of PPI, whereas non-empirical papers were more apt to have a critical eye on PPI. Non-empirical papers presented a broader perspective and offered a discussion of how to measure the impact of PPI in research. Some studies in our review described how PPI enhanced and validated their study and even a cancellation of a RCT [30]. In these cases, PPI had a favorable influence, albeit with varied outcomes. It is important to acknowledge that conducing an unnecessary RCT constitutes misallocation of funding and resources.

Staniszewska et al. discussed the negative impact of PPI in earlier papers and how formalization of more recent publications has become a barrier to critical discussion of the concept [82]. Russel et al. also argued that more research regarding the negative effects of PPI in healthcare research was necessary, as PPI may increase inequality rather that amplifying specific voices and agendas, and calls for a discussion of how to distinguish among measuring, impact, and evaluation [9]. Malterud and Elvbakken expressed their hesitations regarding the intensified use of PPI in healthcare research [83]. Their concerns were that PPI is prioritized at the expense of academic skills, scientific quality, and knowledge outcomes, which may be because of the nature of the papers. Pii et al. also noted a lack of critical reflection on the PPI process, with challenges being only briefly described [8]. Furthermore, Pii et al. stated that some papers reported the use of PPI in the healthcare research as time-consuming. This challenge was supported by Bombard et al. who argued that PPI could be perceived as too time-consuming, burdensome, tokenistic, and disappointing, especially if suggestions were not adopted [84]. Open and constructive discussions to address potential challenges and limitations of PPI in research continues to be warranted.

In our scoping review, we identified gray literature from all five Nordic countries, however more gray literature sources were found from Denmark than other Nordic countries. This discrepancy can be attributed to our research team being based in Denmark. PPI was predominately given consideration at universities and funding bodies. Despite the increasing interest in PPI, we found no obvious collaboration among organizational and institutional stakeholders. Sand et al. described the importance of exchanging views and experiences regarding PPI to enhance its relevance and quality of utilization [2].

The quality of PPI in healthcare research could potentially be improved by establishing national and Nordic consensus for conducting and reporting PPI as they provide guidance and support to researchers enabling to standardized approach to PPI implementation. The UK based NIHR offers an advanced guideline on PPI utilization in healthcare research, and even a detailed calculator of expenses when incorporating PPI [85]. Due to the lack of consensus in the Nordic countries, the integration of PPI is more challenging, despite a generally positive interest and minimal resistance. A recent study from Denmark presented guidelines for researchers discussing methods for integrating PPI [79]. The authors highlighted that a systematic approach to monitoring the extent and impact of PPI is warranted, as a common guideline may improve PPI quality through transparency, consistency and effectiveness in planning, execution and reporting activities.

Despite the growing interest in including PPI in healthcare research, our findings indicate that the impact and meaningful evidence of PPI are still underreported. A European handbook of meaningful patient involvement was published in 2003, and a Canadian instrument was developed to measure meaningful patient and user engagement [86, 87]. A similar instrument has not been developed for the Nordic countries. We found the modified GRIPP2-SF to be valuable for extracting PPI information from the specific research studies due to its comprehensive and user-friendly nature. However, adapting the extraction of PPI information to the Nordic context could convey other aspects beyond the scope of this review. Given the healthcare systems being funded through taxation in the Nordic countries, people involved in research may have different motivations for their participation and contributions to healthcare, e.g., patients might perceive their involvement as a means of contributing back to both the healthcare system and the broader society. Extracting the impact of PPI from the empirical studies in our review was challenging, and as a result, our knowledge of the positive and negative impact of PPI in the Nordic countries remains limited.

### Strengths and limitations

This scoping review is the first to explore and delineate the current state, practice, and impact of PPI in healthcare research across various healthcare domains and patient populations within the Nordic countries. Our comprehensive systematic and gray literature search, which included nine databases with a wide range of search terms, enabled retrieval of a considerable number of references and provided a unique and synergistic review of PPI in the Nordic countries. Using GRIPP2-SF, the included empirical studies were assessed, providing a comprehensive overview of the intent, utilization, and impact of PPI in the Nordic countries. Overall, access to the experiences of PPI in healthcare research was readily available, giving us the added advantage of learning from other Nordic countries. Additionally, empirical papers did not generally describe the specific aims, outcomes, and impact of PPI.

Due to the wide range of PPI terms and the broad scope of our investigation, the search yielded a large number of references with high search sensitivity but low specificity, indicating that while the search retrieved articles of interest, it also included a significant number of articles that were not pertinent to our review.

There were limitations in accessing gray literature from Finland and Iceland, despite our attempts to involve researchers from these two countries, who declined due to limited resources, interest, and funding. The study was skewed due to the investigators’ familiarity with Danish, Norwegian and Swedish language and sparce knowledge of Finnish and Icelandic.

## Implications

The recommendations and future research presented in this scoping review are derived from our findings which aimed to investigate PPI in healthcare research and its impact in the Nordic countries. However, this review posed challenges due to the lack of explicit PPI strategies in healthcare research within these countries. To address this issue, we suggest that each country articulate its approach or policy on PPI in research. This might promote and facilitate a more effective use of PPI to improve overall research outcomes. Collaboration among organizations and institutions, coupled with improved communication, could facilitate a more extensive exchange of experiences and knowledge. This could contribute to the development and assessments of PPI approaches, which are crucial in improving the evaluation of PPI’s impact. PPI could contribute to the improvement of research quality and enhance the relevance and impact of healthcare research, such as improved recruitment and dropout rates [88]. Standardization of PPI approaches could potentially enhance impact within the specific study context and improve methods for measurement of impact and comparison of PPI utilization. However, merely fulfilling PPI requirements mandated by funding bodies may not improve research quality. We suggest, that actively involving patients and the public may result in researchers ensuring that their studies address the needs and priorities of the target population, leading to more impactful and patient-centered research outcomes. Further, enhancing the precision and transparency in describing the recruitment and sampling strategy of people involved could be beneficial. For instance, considering patients’ level of involvement in the research process based on where they are within their treatment trajectory, such as whether they are at the beginning or end of their treatment. PPI highlights the potential for cross-cultural learning and the exchange of best practices in PPI between Nordic countries and the international research community. Future healthcare researchers could benefit from education and guidance on effectively integrating PPI into their research, which could involve learning about various PPI methods and theoretical frameworks, and evaluating the impact of PPI in the context of healthcare research.

## Conclusions

In this scoping review, we examined the trends and practices of PPI in healthcare research across the Nordic countries. We observed a substantial growth in the number of references reporting and addressing PPI in healthcare research, indicating a growing interest in the topic within the Nordic countries. Despite similarities in healthcare systems, there were variations in PPI methodologies, demonstrating its broad application. Despite these variations, there is a shared emphasis on person-centered research practices within the Nordic countries. Given that PPI is a relatively new research approach in the Nordic countries, specific implications have not yet fully emerged. The diverse application of PPI methods and frameworks suggests a lack of established national and international recommendations. This diversity, while enriching the field, also poses challenges in interpreting and synthesizing the findings of our scoping review. Further, it is advisable to consider cost–benefit analyses to rationalize the impact of PPI, so researchers can ensure that PPI efforts are meaningful, efficient, and sustainable, thereby optimizing the positive outcomes while minimizing unnecessary costs.

### Supplementary Information

**Additional file 1.** Example of search strategy in Medline.

## Data Availability

On reasonable request.
