# Supplementary material for: Patient and public involvement in Nordic healthcare research: a scoping review of contemporary practice
Source: Res Involv Engagem. 2023 Aug 30;9:72. doi: 10.1186/s40900-023-00490-x (PMC10466765; doi:10.1186/s40900-023-00490-x)
Supplement: Supplementary file 1 — Additional file 1. Example of search strategy in Medline. [file 40900_2023_490_MOESM1_ESM.pdf]

Supplementary Table 1. Example of search strategy in Medline

Focus area #1	Community Participation/ OR<br>(Citizen* ADJ coalition*) OR<br>(Citizen* ADJ2 collaborat*) OR<br>(Citizen* ADJ2 contribut*) OR<br>(Citizen* ADJ2 cooperat*) OR<br>(Citizen* ADJ2 engag*) OR<br>(Citizen* ADJ2 involve*) OR<br>(Citizen* ADJ2 opinion*) OR<br>(Citizen* ADJ2 participat*) OR<br>(Citizen* ADJ2 role) OR<br>(Citizen* ADJ1 voice*) OR<br>(Client* ADJ coalition*) OR<br>(Client* ADJ2 collaborat*) OR<br>(Client* ADJ2 contribut*) OR<br>(Client* ADJ2 cooperat*) OR<br>(Client* ADJ2 engag*) OR<br>(Client* ADJ2 involve*) OR<br>(Client* ADJ2 opinion*) OR<br>(Client* ADJ2 participat*) OR<br>(Client* ADJ2 role) OR<br>(Client* ADJ1 voice*) OR<br>(Collaborative ADJ inquiry).ti,ab,kw. OR<br>(Communit* ADJ coalition*).ti,ab,kw. OR<br>(Communit* ADJ2 collaborat*).ti,ab,kw. OR<br>(Communit* ADJ2 contribut*).ti,ab,kw. OR<br>(Communit* ADJ2 cooperat*).ti,ab,kw. OR<br>(Communit* ADJ2 engag*).ti,ab,kw. OR<br>(Communit* ADJ2 involve*).ti,ab,kw. OR<br>(Communit* ADJ2 opinion*).ti,ab,kw. OR<br>(Communit* ADJ2 participat*).ti,ab,kw. OR<br>(Communit* ADJ2 role).ti,ab,kw. OR<br>(Communit* ADJ1 voice*).ti,ab,kw. OR<br>(Consumer* ADJ coalition*).ti,ab,kw. OR<br>(Consumer* ADJ2 collaborat*).ti,ab,kw. OR<br>(Consumer* ADJ2 contribut*).ti,ab,kw. OR<br>(Consumer* ADJ2 cooperat*).ti,ab,kw. OR<br>(Consumer* ADJ2 engag*).ti,ab,kw. OR<br>(Consumer* ADJ2 involve*).ti,ab,kw. OR<br>(Consumer* ADJ2 opinion*).ti,ab,kw. OR<br>(Consumer* ADJ2 participat*).ti,ab,kw. OR<br>(Consumer* ADJ2 role).ti,ab,kw. OR<br>(Consumer* ADJ1 voice*).ti,ab,kw. OR<br>(Cooperative ADJ behav*).ti,ab,kw. OR<br>(Expert* ADJ1 experience).ti,ab,kw. OR<br>(Patient* ADJ coalition*).ti,ab,kw. OR<br>(Patient* ADJ2 collaborat*).ti,ab,kw. OR

	(Patient* ADJ2 contribut*).ti,ab,kw. OR<br>(Patient* ADJ2 cooperat*).ti,ab,kw. OR<br>(Patient* ADJ2 engag*).ti,ab,kw. OR<br>(Patient* ADJ2 involve*).ti,ab,kw. OR<br>(Patient* ADJ2 opinion*).ti,ab,kw. OR<br>(Patient* ADJ2 participat*).ti,ab,kw. OR<br>(Patient* ADJ1 partner*).ti,ab,kw. OR<br>(Patient* ADJ2 role).ti,ab,kw. OR<br>(Patient* ADJ2 voice*).ti,ab,kw. OR<br>(Private-public ADJ coalition*).ti,ab,kw. OR<br>(Private-public ADJ1 collaborat*).ti,ab,kw. OR<br>(Private-public ADJ1 cooperat*).ti,ab,kw. OR<br>(Private-public ADJ1 partner*).ti,ab,kw. OR<br>(Public ADJ coalition*).ti,ab,kw. OR<br>(Public ADJ2 collaborat*).ti,ab,kw. OR<br>(Public ADJ2 contribut*).ti,ab,kw. OR<br>(Public ADJ2 cooperat*).ti,ab,kw. OR<br>(Public ADJ2 engag*).ti,ab,kw. OR<br>(Public ADJ2 involve*).ti,ab,kw. OR<br>(Public ADJ2 participat*).ti,ab,kw. OR<br>(Public ADJ2 role).ti,ab,kw. OR<br>(Public ADJ2 voice).ti,ab,kw. OR<br>(Public-private ADJ coalition*).ti,ab,kw. OR<br>(Public-private ADJ1 collaborat*).ti,ab,kw. OR<br>(Public-private ADJ1 cooperat*).ti,ab,kw. OR<br>(Public-private ADJ1 partner*).ti,ab,kw. OR<br>(User* ADJ coalition*).ti,ab,kw. OR<br>(User* ADJ2 collaborat*).ti,ab,kw. OR<br>(User* ADJ2 contribut*).ti,ab,kw. OR<br>(User* ADJ2 cooperat*).ti,ab,kw. OR<br>(User* ADJ2 engag*).ti,ab,kw. OR<br>(User* ADJ2 involve*).ti,ab,kw. OR<br>(User* ADJ2 opinion*).ti,ab,kw. OR<br>(User* ADJ2 participat*).ti,ab,kw. OR<br>(User* ADJ2 role).ti,ab,kw. OR<br>(User* ADJ1 voice*).ti,ab,kw.
Focus area #2	Clinical Nursing Research/ OR<br>exp Health Services Research/ OR<br>exp Research/ OR<br>(Clinical ADJ stud*).ti,ab,kw. OR<br>Research.ti,ab,kw.
Focus area #3	exp Community-Based Participatory Research/ OR<br>Co-research*.ti,ab,kw. OR<br>GRIPP2.ti,ab,kw. OR<br>GRIPP-2.ti,ab,kw. OR<br>(Participatory ADJ design*).ti,ab,kw. OR<br>(Participatory ADJ1 research).ti,ab,kw. OR<br>Peer-research*.ti,ab,kw. OR<br>(Research ADJ involvement).ti,ab,kw.

Focus area #4	exp Scandinavian and Nordic Countries/ OR<br>(Aland ADJ Island*).ti,ab,kw,ia. OR<br>(Aaland ADJ Island*).ti,ab,kw,ia. OR<br>Danish.ti,ab,kw,ia. OR<br>Denmark.ti,ab,kw,ia. OR<br>Finland.ti,ab,kw,ia. OR<br>Finnish.ti,ab,kw,ia. OR<br>(Faroe ADJ Island*).ti,ab,kw,ia. OR<br>Greenland*.ti,ab,kw,ia. OR<br>Iceland*.ti,ab,kw,ia. OR<br>(Nordic ADJ countr*).ti,ab,kw,ia. OR<br>(North ADJ atlantic*).ti,ab,kw,ia. OR<br>Norway.ti,ab,kw,ia. OR<br>Norwegian*.ti,ab,kw,ia. OR<br>Scandinavia*.ti,ab,kw,ia. OR<br>Sweden.ti,ab,kw,ia. OR<br>Swedish.ti,ab,kw,ia.

A 1 AND 2 AND 4 = 875

B 3 AND 4 = 118

C (1 AND 2 AND 4) OR (3 AND 4) = 956

-------- notes --

The ADJ operators finds two terms next to each other in the specified order. The ADJ1 operators finds two terms next to each other in any order. The ADJ2 operator finds terms in any order and with one word (or none) between them. The ADJ3 operator finds terms in any order with two words (or fewer) between them. The ADJ4 operator finds terms in any order and with three words (or fewer) between them, and so on.

Supplementary Figure 1 Medline, search performed on September 16th, 2021
